# Comparison of computer simulations and clinical treatment results of magnetic resonance‐guided focused ultrasound surgery (MRgFUS) of uterine fibroids

**DOI:** 10.1002/mp.15263

**Published:** 2022-03-02

**Authors:** Mikko Hyvärinen, Yuexi Huang, Elizabeth David, Kullervo Hynynen

**Affiliations:** ^1^ Sunnybrook Research Institute Toronto Ontario Canada; ^2^ Department of Medical Biophysics University of Toronto Toronto Ontario Canada

**Keywords:** clinical results, computer simulation, HIFU, MRgFUS, uterine fibroids

## Abstract

**Purpose:**

Magnetic resonance‐guided focused ultrasound surgery (MRgFUS) can be used to noninvasively treat symptomatic uterine fibroids by heating with focused ultrasound sonications while monitoring the temperature with magnetic resonance (MR) thermometry. While prior studies have compared focused ultrasound simulations to clinical results, studies involving uterine fibroids remain scarce. In our study, we perform such a comparison to assess the suitability of simulations for treatment planning.

**Methods:**

Sonications (*N* = 67) were simulated retrospectively using acoustic and thermal models based on the Rayleigh integral and Pennes bioheat equation followed by MR‐thermometry simulation in seven patients who underwent MRgFUS treatment for uterine fibroids. The spatial accuracy of simulated focus location was assessed by evaluating displacements of the centers of mass of the thermal dose distributions between simulated and treatment MR thermometry slices. Temperature–time curves and sizes of 240 equivalent minutes at 43°C (240EM_43_) volumes between treatment and simulation were compared.

**Results:**

The simulated focus location showed errors of 2.7 ± 4.1, −0.7 ± 2.0, and 1.3 ± 1.2 mm (mean ± SD) in the anterior–posterior, foot–head, and right–left directions for a fibroid absorption coefficient of 4.9 Np m^–1^ MHz^–1^ and perfusion parameter of 1.89 kg m^–3^ s^–1^. Linear regression of 240EM_43_ volumes of 67 sonications of patient treatments and simulations utilizing these parameters yielded a slope of 1.04 and a correlation coefficient of 0.54. The temperature rise ratio of simulation to treatment near the end of sonication was 0.47 ± 0.22, 1.28 ± 0.60, and 1.49 ± 0.71 for 66 sonications simulated utilizing fibroid absorption coefficient of 1.2, 4.9, and 8.6 Np m^–1^ MHz^–1^, respectively, and the aforementioned perfusion value. The impact of perfusion on peak temperature rise is minimal between 1.89 and 10 kg m^–3^ s^–1^, but became more substantial when utilizing a value of 100 kg m^–3^ s^–1^.

**Conclusions:**

The results of this study suggest that perfusion, while in some cases having a substantial impact on thermal dose volumes, has less impact than ultrasound absorption for predicting peak temperature elevation at least when using perfusion parameter values up to 10 kg m^–3^ s^–1^ for this particular array geometry, frequencies, and tissue target which is good for clinicians to be aware of. The results suggest that simulations show promise in treatment planning, particularly in terms of spatial accuracy. However, in order to use simulations to predict temperature rise due to a sonication, knowledge of the patient‐specific tissue parameters, in particular the absorption coefficient is important. Currently, spatially varying patient‐specific tissue parameter values are not available during treatment, so simulations can only be used for planning purposes to estimate sonication performance on average.

## 1 INTRODUCTION

Uterine fibroids (UF), also known as leiomyomata, are commonly found benign growths of the uterus originating from smooth muscle cells. Fibroids occur in over 70% of women,^1^ and approximately 25% of the fibroid bearing population has symptomatic fibroids,^2^ which can degrade the quality of life. The current standard of care treatment for symptomatic UFs includes medical and surgical options which can range from drug therapy, myomectomy, hysterectomy, and embolization.

Magnetic resonance‐guided focused ultrasound surgery (MRgFUS) is a relatively new treatment modality that is used to treat fibroids^3^ as well as a variety of other conditions.^4^ During the treatment of fibroids, ultrasound is focused on a region within the fibroid. The ultrasound energy is absorbed by the tissue, causing a temperature rise. If the temperature rise is sufficiently high and maintained long enough, the result is thermal coagulative necrosis. Once a sufficiently large volume of the fibroid is ablated, the fibroid shrinks over a period of months^5^ and the patient is alleviated of her symptoms. Magnetic resonance (MR) guidance allows pre‐treatment localization of anatomy, temperature monitoring during the treatment, as well as an immediate assessment of the treatment outcome by visualizing the non‐perfused volume with gadolinium (Gd) contrast.^6^, ^7^

Advantages of MRgFUS in UF treatments over current standard of care treatments are that MRgFUS is non‐invasive and the recovery time of patients is much shorter. Fertility can also be preserved after MRgFUS treatments.^8^ Focused ultrasound surgery can also be performed with US guidance,^9^ but without the same level of real‐time monitoring and control that is possible with MR thermometry.

While MRgFUS treatments have shown great success^2^, ^10^ and are continuously being improved,^2^ factors currently hindering the widespread adoption of this treatment modality as a clinical standard for UFs include the long duration of treatments,^11^, ^12^ requiring expensive MRI time, as well as patient screening criteria and difficulty heating certain types of fibroids limiting the treatment envelope.^13^ The treatment envelope refers to the types and locations of fibroids that can be effectively treated with MRgFUS. Tissues outside of the target volume within the beam path accumulate heat which contributes to long treatment times as it is necessary to wait for these structures to cool prior to starting the next sonication.^14^ Furthermore, when treating fibroids that are difficult to heat, non‐target heating can occur to such an extent that the achievable treatment volume is limited by the need to protect adjacent normal tissue. Heating profiles in the tissue and the extent to which non‐target heating contributes depends on the geometry of the transducer and patient, patient‐specific tissue composition, and operator adjustable sonication parameters and strategies. An example of a sonication strategy is provided in a study^15^ performed using a single‐element transducer and a phantom which suggests that algorithms used to determine the order of locations of the acoustic focus within a grid could reduce the extent of near‐field heating.

To overcome lengthy treatment times, phased array transducers, which are capable of electronically steering the acoustic focus, and sonication strategies have been developed. For example, with a phased array transducer it is possible to perform volumetric ablation,^16^, ^17^ described in further detail in Section 2, by rapidly switching between fields having multiple foci or by steering the acoustic focus rapidly and repetitively along trajectories within a volume. Volumetric ablation is a treatment strategy that is currently being used clinically to shorten treatment times.

To illustrate the current stage of treatment planning in the context of volumetric ablation of UFs, the Philips Sonalleve system shall be referred to as an example. Treatments on the Sonalleve are performed by placing sonication cells within the fibroid, one after another until a sufficiently large volume of the fibroid is ablated. These sonication cells consist of circular trajectories of focal points about which the focus is scanned repeatedly.^17^ The resulting ellipsoidal‐shaped region of ablative temperatures shall be referred to as the thermal focus. The first time a selected target depth is sonicated with the transducer at a particular orientation, a low power test sonication is performed to verify that the thermal focus is at the intended depth location. If necessary, adjustments are made and the procedure is repeated. The sonication parameters of the test sonication are designed to create temperatures low enough not to cause any damage to tissues, while being sufficiently large to be measured using MR thermometry. After these tests, the sonication cells are placed and sonicated one after another. Sonication parameters are determined during the treatment based on outcomes of previous sonications. Typically, it is necessary to wait up to several minutes between sonications for the tissue in the near field of the transducer to cool.

A major factor contributing to lengthy treatment times is the high rate of incomplete sonications (sonications that are not completed as intended). For example, some centers report that roughly one out of four sonications performed with the Sonalleve are aborted by the patient, operator, or automatic system safety checks.^18^ Aborted sonications typically occur when excessive near‐field or far‐field heating develops before the desired temperature for ablation is reached at the focus, causing additional waiting time. The rate of incomplete volumetric feedback sonications on the Sonalleve is reported to be as high as 70%.^12^ Another contributor to long treatment times in current clinical treatments is that parameters such as the order of the treatment cells and the cooling time in between sonications are most likely not optimal.

The use of computer models to calculate acoustic fields, temperature, and thermal dose distributions to assist in guiding patient treatments and developing treatment protocols in this publication is referred to as model‐based treatment planning (MBTP). Improvements in MBTP could help better predict the temperature and thermal dose prior to sonication, prevent aborted sonications, and enable the optimization of sonication parameters and the order of sonication cells^19^ to shorten treatment time.

Two factors that make fibroids difficult to heat and thus limit the treatment envelope are thought to be perfusion^20^, ^21^, ^22^ and low ultrasound absorption.^23^ The tissue‐specific perfusion and ultrasound attenuation parameters are poorly quantified and can lead to variable outcomes and treatment inefficiencies.^24^ However, a small number of quantitative estimates of in vivo UF perfusion are available in the literature including a feasibility study using an analytic technique^25^ and a study involving one patient case exhibiting low clinical heating.^26^ Similarly, ex vivo estimates of UF attenuation are available in several studies,^27^, ^28^, ^29^ (while in vivo values remain scarce in the literature).^30^, ^31^ By gaining information about the particular patient‐specific tissue parameters that limit and hinder the heating of a fibroid during a particular sonication, it could be possible to select more optimal sonication parameters for such a sonication. This could aid in attaining heating of the fibroid and play a role in expanding the treatment envelope.

Acoustic^32^, ^33^ and thermal^33^ simulations have been run in UF patient geometries in earlier studies for device development and treatment planning purposes. Suomi et al.^26^ used acoustic simulations in a patient treatment geometry for a case exhibiting low clinical heating to study the effects of tilting the transducer. However, while results of acoustic simulations have been compared to phantom experiments^32^ and the results of thermal simulation have been compared to MR‐thermometry data from animal experiments,^34^ to the authors’ knowledge, a comparison of results of such simulations has not yet been performed with respect to MR‐thermometry UF patient data. In this study, we perform a comparison of simulation results with clinical MR‐thermometry data acquired from UF treatments as a first step with the longer‐term goal of furthering applications of the simulations for MBTP. For example, improved MBTP may have potential to reduce treatment times, make treatments safer, and expand the treatment envelope and move us toward greater utilization of MRgFUS in the treatment of UFs. In performing this comparison, we characterize the ability of the model to predict the location of the thermal focus, magnitude of peak temperature rise, as well as sizes of focal thermal dose threshold volumes (regions of temperature rise high enough to be clinically relevant) averaged over sonications within as well as throughout patient treatments using several constant values of tissue parameters. In addition to gaining an understanding of the effect sizes of varying perfusion and absorption parameters, we investigate the role these parameters may have in explaining clinically observed variability in temperature rise from patient to patient as well as from location to location within a patient.

## 2 MATERIALS AND METHODS

### 2.1 Description of patient population

A total of seven patient cases who were treated for symptomatic UFs at Sunnybrook using the Sonalleve were retrospectively analyzed and compared to computer simulations. UF patient treatment data involving the Sonalleve system as opposed to other commercial clinical systems was included in this study based on availability of patient treatment data.

### 2.2 Description of clinical device and patient treatments

The Sonalleve consists of a 256‐element random spherical sparse ultrasound transducer array immersed in mineral oil within an MRI tabletop. The transducer can move in three spatial directions as well as tilt. The geometric focal length of the transducer used for these patient treatments was 14 cm. The frequencies available for clinical sonications are approximately 1.20 and 1.44 MHz and can be chosen for each sonication by the operator.

The treatments on the Sonalleve utilize volumetric ablation^17^ due to its energy efficiency.^11^, ^35^ A sonication is performed on a construct called a treatment cell, which consists of points over which the acoustic focus is electronically steered lying on axially concentric circular trajectories intended to produce an ellipsoidally shaped region of ablated tissue. Treatment cells are placed within treatment cell clusters, where a treatment cell cluster is a term describing treatment cells confined to a layer defined by fixing degrees of freedom of the transducer, e.g., depth and/or tilt angle(s). During the treatment, a cell size and type are selected, with possible cell sizes including 4‐, 8‐, 12‐, 14‐, and 16 mm‐diameter ellipsoids consisting of 1, 2, 3, 4, and 4 concentric circular trajectories, respectively. Treatment cell types include so‐called regular cells in which switching times from one trajectory to the next are preset^17^ and feedback cells^36^ in which the trajectory switching times are set to occur based on the feedback from MR thermometry involving temperature and/or thermal dose conditions.

The temperature distribution within the focal volume is monitored by three coronal slices and one sagittal slice. The coronal slice thickness and gap are 7.0 and 0.0 mm, respectively, thus covering a continuous volume (having a length of 21 mm in the depth direction) containing the treatment cell position. A near‐field and far‐field slice, whose locations are defined by the clinician, are also used for temperature monitoring. The MR thermometry technique used to monitor the temperature is proton resonance frequency shift thermometry.^37^ The temperature is monitored during each sonication and the monitoring is stopped before the beginning of the next sonication. Additional MR‐thermometry parameters include repetition time (TR) of 25.17 ms, echo time (TE) of 16.00 ms, image flip angle (FA) of 18.00 deg, slice thickness of 7 mm, field of view (FOV) of 400 by 300 mm, acquisition matrix of dimension 192 × 143, and scan percentage of 99.3. A multishot echo planar imaging (EPI) sequence with an EPI‐factor of 11 utilizing these parameters was used to obtain thermal maps with dynamic (6 slices) acquisition time of approximately 2.57 s resulting in each slice having reconstructed pixel spacing of 2.083 by 2.083 mm. The temperature maps were embedded in a 192 × 192 matrix with pixels outside of the FOV being assigned a constant baseline temperature.

T2‐weighted treatment planning images are acquired in order to localize patient anatomy prior to focused ultrasound treatment. The patient lies prone on the MR‐tabletop and is acoustically coupled to the transducer within the tabletop by a gel pad that sits in water on top of a thin mylar membrane. MR‐imaging scans used to look for the presence of bubbles within the acoustic coupling media are also performed when deemed necessary to ensure that there are no bubbles present which could interfere with the treatment. However, sometimes additional scans are acquired, including repeated T2‐weighted treatment planning scans during treatment if the patient has moved.

After anatomical scans have been completed, using the treatment planning system graphical interface, the clinician then places treatment cells. The transducer is positioned by the system using a combination of electronic and/or mechanical steering based on the chosen location and orientation of the treatment cell. A low‐power test sonication is performed to verify target accuracy on the first treatment cell of each cluster, but may be performed on other cells as well. The treatment planning system then calculates a spatial heating offset based on the temperature distribution resulting from the test sonication measured by MR‐thermometry. The transducer is then moved in an attempt to counteract the heating offset by an amount termed the misregistration correction. The misregistration correction can also be defined manually by the clinician. Multiple therapeutic sonications (sonications intended to cause thermal ablation) are often performed following a test sonication yielding a satisfactory result. Thermal dose distributions are calculated from the MR‐thermometry temperature maps, which are used along with the temperature distributions to help make treatment parameter decisions, e.g., to define the position, size, and power level of a following treatment cell. A sonication may be stopped by the patient or clinician administering the treatment, as well as by the treatment system software.

In order to assess the outcome at the end of the treatment, the non‐perfused volume resulting from the ablative procedure is visualized by acquiring Gd contrast‐enhanced post‐treatment T1‐weighted images.

### 2.3 Phantom validations

#### 2.3.1 Phantom experiment

**FIGURE 1:**
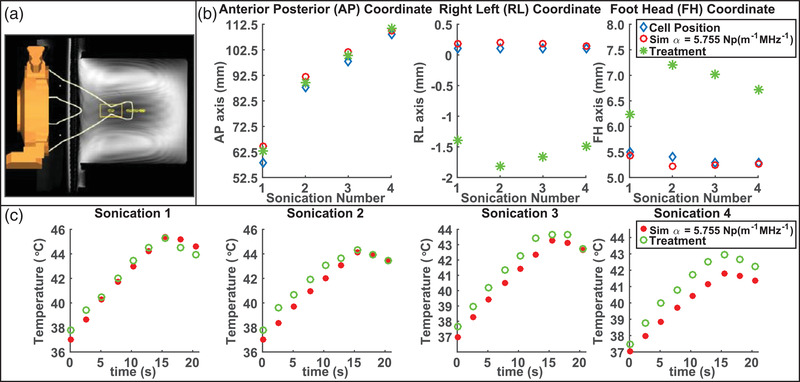
(a) Phantom experiment geometry with depths of treatment cell positions (targets) shown as they appear on the treatment planning system. (b) The anterior–posterior (AP), right–left (RL), and foot–head (FH) coordinates of the location of the simulated and experimental thermal dose distribution centers of mass as well as the treatment cell position (target). (c) Peak temperature curves of the simulated and experimental MR‐thermometry sagittal slices as a function of time

A phantom experiment was performed to validate the simulation model in a phantom geometry, as well as validate the calculation used to determine transducer element phases for the simulations via backpropagation using the parameters in the log together with parameters in the treatment planning system configuration files (see Figure S1 and the text in Supporting Information). The phantom used in the experiment was provided by Philips Sonalleve for purposes of performing quality assurance prior to clinical treatments and to the best of the authors’ knowledge consists of an undisclosed proprietary material. No gel pad was used in the phantom experiment and the phantom was placed sitting in water directly on top of the membrane covering the oil that the transducer is immersed in. Sonications were performed at several different depths illustrated in Figure 1.

#### 2.3.2 Phantom simulations

The simulation pipeline consisting of acoustic, thermal, and MR thermometry simulation (described in the following section) was validated by implementing it on sonications performed in the phantom experiment. Sonications were simulated at four different depths in the phantom. The notable difference between the simulation workflow for a phantom sonication versus a patient sonication was that there were only two different media (oil and phantom material) in the phantom simulation model and thus for each phantom sonication the velocity used to determine the pressure was calculated using only one contour (the phantom‐membrane contour separating the phantom from the oil in the layered model). The velocity used to determine the pressure within the phantom was then calculated on an area of this contour sufficiently large to intersect with the normal vectors of transducer elements. The speed of sound of the phantom used in the simulations was 1540 m s^–1^. The attenuation of the phantom used in the simulations was 5.755 Np m^–1^ MHz^–1^. While absorption estimates of phantoms have been demonstrated in the literature,^38^ the absorption coefficient of the phantom in the simulations was taken to be the value of attenuation as this is consistent with the simulation methodology used in the patient treatments. The values of these parameters provided by the vendor (Philips Sonalleve) for the phantom are 1540 ± 10 m s^–1^ and 0.5 ± 0.05 Db cm^–1^ MHz^–1^. A parameter value of 1050 kg m^–3^ from the configuration was used for the density of the phantom as the density of the phantom was reported as unknown by the vendor (Philips Sonalleve). The thermal conductivity and heat capacity of the phantom are unknown and are assumed to be values used for muscle in simulations, namely 0.4975 W m^‐1^ K^‐1^ and 3565 J kg^–1^ K^–1^. The perfusion parameter in the phantom simulations was set to zero as the phantom experiment did not have perfusion.

The thermal conductivity of the phantom was estimated by using a technique similar to that of Zhang et al.^39^ for five phantom sonications for purposes of verifying that the thermal conductivity parameter used in the phantom simulations is valid. While theoretical background of the technique is explained elsewhere,^40^ the implementation will be described briefly. Temperature rise data from the central coronal MR‐thermometry slice acquired at timepoints during cooling after sonication is fit to a two‐dimensional spatial Gaussian distribution of the form

(1) $$G\left(x_{1},x_{2}\right)\;=\;ae^{-\frac{{\left(x_{1}-b_{1}\right)}^{2}}{2\sigma^{2}}}e^{-\frac{{\left(x_{2}-b_{2}\right)}^{2}}{2\sigma^{2}}}.$$

The thermal conductivity estimate is calculated via

(2) $$\kappa \;=\;\left(\frac{\rho C}{2}\right)\frac{d\sigma^{2}}{dt},$$

where ρ and *C* denote the density and the heat capacity, and the value of $d\sigma^{2}/dt$ is taken to be the slope of the regression line obtained by fitting σ^2^ as a function of time. The two‐dimensional Gaussian fitting was performed with MATLAB R2015a using lsqnonlin with variables *a*, *b*_1_, *b*_2_, and σ all being fitting parameters. The initial values for the fitting were defined as peak temperature for *a*; location of peak temperature for the pair of variables *b*_1_ and *b*_2_; and a value of 1 voxel for σ. The cooling intervals were defined to start at the slice following peak temperature and consisting of 16 slices were 38.6 s long. This implementation resulted in a median value of 0.557 W m^–1^ K^–1^, a range of 0.529–0.773 W m^–1^ K^–1^, and a mean  ± SD of 0.628 ± 0.121 W m^–1^ K^–1^. These results justify using the thermal conductivity value of muscle in the phantom simulations considering that it is very close to the median estimated value and furthermore that varying thermal conductivity within the range of estimates is unlikely to have a substantial effect on the results of Figure 1.

### 2.4 Description of simulations

A total of *N* = 67 sonications were simulated using acoustic and thermal models and compared to treatment results. The sonications were selected from the treatment data of seven UF patients included in this simulation study. While sonications are performed on treatment cells, more than one sonication potentially having different parameters (e.g., power) can be performed on the same treatment cell. The treatment cell sizes on which these sonications were performed ranged from 4 to 16 mm in diameter. Lengths of treatment cells having diameters of this range were estimated using the treatment planning system graphical user interface and ranged from approximately 10 to 35 mm. For details about sonication parameters, see Table S1.

#### 2.4.1 Simulation geometry, patient anatomy, and coregistration

The size of the transducer elements and their coordinates were obtained from the configuration file of the treatment planning system. These parameters were then used to construct the surface geometry of the transducer at approximately six points per wavelength using the speed of sound of (mineral) oil defined in the configuration files of the treatment planning system. This surface geometry was used in the acoustic model as described in the section titled ‘‘Workflow of a Simulated Sonication.’’

Patient anatomy for simulation geometries was derived from T2 weighted MR treatment planning images by segmenting the images manually.^33^ The anatomical layers that were segmented manually are the fat, abdominal muscle, and the tissues posterior of the abdominal muscle consisting generally of uterus and fibroid taken together. The skin was segmented by creating a contour anterior of the boundary between the skin and the fat by adding 2 mm. The gel pad was segmented by adding 15 mm to the outer boundary of the skin. In some areas where MR artifacts could visually be identified, interpolations were performed. In cases where the MR treatment planning image set did not cover a sufficient amount of the skin–fat layer, extrapolations were performed. The medium anterior of the gel pad was modeled using the parameters of mineral oil in the configuration files of the treatment planning system. The patient geometry that was derived from treatment planning images was used in both acoustic and thermal simulations. In cases of multiple T2‐weighted scans, the scan acquired most recently before a given sonication was used to derive the simulation geometry for the sonication.

The transducer geometry and patient anatomy were coregistered to the coordinate system of the scanner. The parameters from the treatment planning system log files were used to determine the position of the transducer for each sonication in the scanner coordinate system of the treatment planning system. The misregistration correction was incorporated into the positioning of the transducer in the simulations: According to the log files, the transducer position was shifted by the amount indicated by the misregistration correction so the simulated position of the transducer was also shifted in an effort to do the modeling with the transducer in the same position as in the treatments, e.g., if the transducer was shifted anteriorly by the amount due to the misregistration correction then so was the simulated position of the transducer. The patient anatomy was coregistered to the scanner coordinate system by an affine transformation derived from coordinates of points in both image and scanner coordinate systems. The coordinates’ tool of the treatment planning system was used to determine the values of the coordinates while in the orthogonal and/or transducer view.

#### 2.4.2 Description of acoustic model

The Rayleigh–Sommerfeld integral is a widely accepted model used for the propagation of ultrasound. As described in the literature,^32^, ^34^, ^41^, ^42^, ^43^ it can be used to model the propagation of ultrasound through layered media, taking into account attenuation, reflection, and refraction. Fan's model^43^ uses the Rayleigh integral and the numerical approach by Zemanek^42^ in a two‐step procedure. First, the velocity at a boundary between two layers is calculated using the velocity of the previous boundary with the first boundary being the transducer, via

(3) $$u\;\left(r\right)=\;\frac{ik}{2\pi }\int_{S}u_{src}\left(r^{\prime }\right)\frac{e^{-ik_{c}R}}{R}\left(1-\;i\frac{1}{kR}\right)T\cos\left(\theta_{t}\right)ds,$$

where $k_{c}=\;k-i\alpha$ is the complex wave number of the medium with attenuation coefficient α, $r^{\prime }$ is the position vector on the surface element having a velocity $u_{src}$ and area ds, *r* is the position vector on the surface element whose velocity is to be calculated, $R\;=\;|r-r^{\prime }|$. T is the particle velocity transmission coefficient calculated as

(4) $$T\;=\frac{2\rho_{1}c_{1}\cos\left(\theta_{i}\right)}{\rho_{2}c_{2}\cos\left(\theta_{i}\right)+\rho_{1}c_{1}\cos\left(\theta_{t}\right)},\;\;$$

where $\rho_{1}c_{1}$ and $\rho_{2}c_{2}$ denote the characteristic acoustic impedances of the media on the incident and transmitted sides, respectively. The angles $\theta_{i}$ and $\theta_{t}$ respectively are the incidence and transmission angles referenced to the normal vector of the surface on which the velocity is being calculated. Second, the pressure within a layer is calculated using the velocity at its anterior boundary via

(5) $$p\left(r\right)=\frac{\mathrm{ik}\rho c}{2\pi }\;\int_{S}\frac{e^{-ik_{c}R}}{R}\mathrm{uds},$$

where r is the position vector of the point at which the pressure is to be calculated, and ρ and c respectively are the density and the speed of sound of the medium. For points that are very close to the boundary, the pressure field was calculated via

(6) $$p\left(r\right)=\frac{ik\rho c}{2\pi }\;\int_{S_{0}}\frac{e^{-ik_{c}R}}{R}uds-\;\frac{k}{k_{c}}\rho c\left|u\right|\left[e^{-ik_{c}\varepsilon }-1\right]|\begin{array}{c} S_{\varepsilon } \end{array},$$

where the surface S is divided into $S_{0}$ for R>ε and $S_{\varepsilon }$ for R≤ε, for $\varepsilon \;=\;\sqrt{ds/\pi }$.^33^

Results of acoustic simulations utilizing the Rayleigh integral have been compared with acoustic fields obtained from experiments in multiple studies,^34^, ^35^, ^43^, ^44^, ^45^, ^46^, ^47^, ^48^ including fields generated by a single element transducer in layered media,^43^ electronically steering the focus of a flat phased array,^48^ and by applying various phase distributions on phased arrays including a 256‐element spherically curved sparse array.^47^ An experimental validation of Rayleigh simulation methodology utilizing a 256‐element phased array transducer yielded good geometric agreement in intensity profiles.^47^.

#### 2.4.3 Description of thermal and thermal dose models

The Pennes bioheat transfer equation is widely used throughout the literature to model temperature distributions.^33^, ^49^ Using the absorbed power density Q(r,t) as the heating source term, the temperature distribution resulting from sonication is calculated according to

(7) $$\begin{array}{ccc} \rho C\;\frac{\partial T\left(r,t\right)}{\partial t} & = & \;\nabla \cdot \left[\kappa \nabla T\left(r,t\right)\right]-\;\omega_{b}\rho_{b}C_{b}\left[T\left(r,t\right)-T_{b}\right]\\ & & +Q\left(r,t\right). \end{array}$$

The first and second terms on the right side of the equation describe the heat transfer by thermal conduction and blood perfusion, and the third describes the contributions of the heat source. In this equation, ρ, C, and κ denote the density, heat capacity, and thermal conductivity of the tissue, respectively. The parameters $\omega_{b}$, $\rho_{b}$, and $C_{b}$ denote the perfusion rate, density, and heat capacity of the blood, respectively. For convenience, as literature values of perfusion are often defined in terms of kg m^–3^ s^–1^, to accommodate these units the parameter ω shall be defined as $\omega \;=\;\omega_{b}\rho_{b}$. The parameters ρ_b_ and C_b_ were set to have values of 1050 kg m^–3^ and 3850 J kg^–1^ K^–1^, respectively. The absorbed power density Q can be calculated from the pressure p via $Q\;=\alpha p^{2}/\rho c\;,$ where α and c are the absorption coefficient and speed of sound of the medium, respectively.

To predict ablated volumes, one may use the Dewey–Sapareto thermal dose formalism,^50^, ^51^ which is defined as

(8) $$t_{43}=\;\sum_{t\;=\;0}^{t_{\mathrm{final}}}R^{43-T}\Delta t,\;\;\;\text{with}\;R\;\;=\;\;\left\{\begin{array}{c} 0.25\;\;\;\;\text{for}\;T<43^{\circ }C\\ 0.5\;\;\;\;\;\;\;\text{for}\;T>\;43^{\circ }C \end{array}\right.,\;$$

where *T* is the time‐averaged temperature during a sufficiently small time interval Δt and $t_{43}$ denotes equivalent minutes at $43^{\circ }C$. In the simulations, the initial body temperature was set to 37°C which was also used as the initial temperature value for calculating the thermal dose.

#### 2.4.4 Workflow of a simulated sonication

For each simulated sonication, the velocity and pressure distributions were calculated using the layered model described earlier, which was then used to obtain the absorbed power density distribution for one target focal point on each circular trajectory. The surface velocities on the transducer elements were initially set to have magnitude 1, and taking into account beam steering, the phases of the velocities on the transducer were calculated using a speed of sound value of 1540 m s^–1^ found in the treatment planning system configuration file, by back‐propagating from a target point calculated by using treatment parameters of the treatment planning system log and configuration files. The absorbed power density fields of the remaining points on the circular trajectory were obtained by rotation. For example, given a treatment cell with 12 mm diameter having three circular trajectories, the absorbed power density would be calculated for three points and those of the remaining points are obtained by rotation. The acoustic simulations were computed using numerical integration (discretization strategy reported in the Supporting Information) with velocity and pressure simulations calculated on GPU (Tesla C2070) and CPU (2 processors, Intel Xeon CPU E5‐2630 v3 at 2.40 GHz) implementations, respectively. To take into account the acoustic power used to drive the transducer, the resulting absorbed power density fields were scaled based on the nominal power value in the treatment planning system log files by multiplying by

(9) $${\left|u_{0}\right|}^{2}=\frac{P_{\mathrm{nominal}}}{\frac{1}{2}\rho_{\mathrm{oil}}c_{\mathrm{oil}}A_{\mathrm{Transducer}}}\;.$$

The tissue parameters used can be found in Table 1. The pressure and absorbed power density fields were calculated using three values of fibroid absorption (described below). The gel pad was modeled having parameters of water.

**TABLE 1:** Parameters of media used in simulations

**Parameter∖Medium**	**Mineral Oil** **^a^**	**Water** **^b^** **(gel pad)**	**Skin** **^b^**	**Fat** **^b^**	**Muscle** **^b^**	**Uterus/fibroid**
*c* (m s^−1^)	1426	1500	1645	1445	1569	1614^b^
ρ (kg m^−3^)	1070	1000	1200	921	1138	1052^b^
α (Np m^−1^ MHz^−1^)	0	2.88×10^−4^	40.0	7.0	9.0	1.2^c^, 4.9^d^, 8.6^b^, ^e^
ω_b_ (s^−1^)	N/A	0	5.83×10^−4^	5.0×10^−4^	3.97×10^−4^	1.8×10^−3^ ^b^, ^f^
κ (W m^−1^ K^−1^)	N/A	0.615	0.3766	0.248	0.4975	0.5^b^
*C_p_* (J kg^−1^ K^−1^)	N/A	4180	3410	2490	3565	3434^b^

^a^ 1426 m s^−1^ and 1070 kg m^−3^ are values from treatment planning system configuration.

^b^ Ellens and Hynynen.^33^

^c^ Siddiqi et al.^52^

^d^ Average of 1.2 and 8.6.

^e^ Within a subset of six sonications, fibroid absorption was varied to take on additional values as described in more detail in Section 2.4.5.

^f^ Within a subset of 22 sonications, fibroid perfusion was varied to take on additional values as described in more detail in Section 2.4.5.

The temperature rise resulting from the sonication was calculated on a Tesla C2070 graphics processing unit (GPU) using a finite difference time domain solver implementation similar to the one described by Ellens and Hynynen^33^ written in C/C++ using Compute Unified Device Architecture (CUDA). Practically, with the current implementation the combined acoustic and thermal simulation of a sonication with one set of parameters is on the order of hours possibly taking up to a day or more.

In order to be able to make a meaningful comparison of simulated temperature distributions to the clinical MR thermometry slices, the simulated temperature distribution was processed into simulated MR‐thermometry slices as described by Enholm et al.^36^ The slices at each timepoint were obtained from the simulated temperature distribution at the particular timepoint without temporally averaging the simulated temperature distribution as a function of time. The sampling interval of the simulated MR‐slices was set to 2.57 s to coincide with the sampling interval of slices acquired during treatments. The voxel dimensions of the slices were set to 2.083 by 2.083 by 7 mm to match the ones used in the clinical slices. For each sonication, a simulated coronal stack and sagittal slice were placed at the treatment cell position to approximately match the location of slices of clinical patient treatment. While there was some uncertainty regarding the relative positioning of the transducer, MR‐thermometry slices, and treatment cell position relative to each other involving the misregistration correction, the effects of this uncertainty on the results of interest were not substantial enough to alter the key findings. This is based on an estimate obtained by incorporating an additional set of simulated MR‐thermometry slice positions into the analysis of simulated MR‐thermometry data in which the coronal stack and sagittal slice were placed at the treatment cell position with the misregistration correction value added to it (see Figures S9, S10, and S11). More detailed information involving the calculations of absorbed power density, thermal simulations, and MR‐thermometry simulation are provided in the Supporting Information.

#### 2.4.5 Description of simulation datasets regarding variation of tissue parameters

To evaluate simulation accuracy compared to treatment results, all 67 sonications selected to be simulated in this study were simulated using three values of fibroid absorption namely 1.2,^52^ 8.6,^28^, ^32^, ^33^ and 4.9 (average) Np m^–1^ MHz^–1^ while holding fibroid perfusion at a constant value of 1.89 kg m^–3^ s^–1^.^33^ Unless otherwise stated, the results throughout this paper utilized this value of fibroid perfusion.

To perform exploration of the effects of perfusion a smaller subset of sonications was selected. To study perfusion effects, varying levels of fibroid perfusion up to 100 kg m^–3^ s^–1^ were simulated for *N* = 22 sonications from treatments of three patients (Figure S5). These sonications were a subset of the 67 sonications. *N* = 15/22 sonications (6 of which were performed in treatment of Patient 2, and 9 in treatment of patient 6) were simulated with an absorption value of 8.6 Np m^–1^ MHz^–1^ and perfusion values of 1.86, 10, and 100 kg m^–3^ s^–1^. *N* = 5/22 sonications (all 5 of which were from the treatment of patient 3) were simulated with absorption values of 8.6 and 1.2 Np m^–1^ MHz^–1^, and for both absorption values perfusion was varied to take on values of 1.86, 10, and 100 kg m^–3^ s^–1^. The remaining *N* = 2/22 sonications were from the treatment of Patient 2, and were simulated with an absorption value of 1.2 Np m^–1^ MHz^–1^ and perfusion values of 1.86 and 10 kg m^–3^ s^–1^.

To further study absorption, simulations using additional fibroid absorption values of 6.8, 9.8, 11.0, 13.3, 18.0 Np m^–1^ MHz^–1^,^30^, ^31^ while holding fibroid perfusion at a constant value of 1.89 kg m^–3^ s^–1^ were run for *N* = 6 sonications for which a fibroid absorption value of 8.6 Np m^–1^ MHz^–1^ did not show a high degree of heating when compared to patient treatment results. As fibroid absorption was varied, fibroid attenuation was also varied to take on the same value as absorption. The anterior–posterior (AP) coordinates of the treatment cell positions (depth) of these sonications spanned from 66.7 mm to 79.6 mm. These sonications were performed in the treatments of Patient 2 (*N* = 2), patient 4 (*N* = 1), patient 6 (*N* = 2), and patient 7 (*N* = 1).

### 2.5 Patient treatment sonication data workflow

For each sonication, MR‐thermometry slices are calculated and processed by an algorithm pipeline of the sonalleve system from phase map acquisitions. In an effort to remove noise and artifacts in regions beyond the focal volume of the patient treatment and experimental phantom MR‐thermometry slices, for each sonication a region containing the focal volume was first defined manually, and then that region was cropped out of all the MR‐thermometry slices associated with the sonication. The cropped regions of the patient treatment and experimental phantom sonication MR‐thermometry slices were used for the calculations described in this and the following section. This cropping procedure was not performed for the simulated MR‐thermometry slices.

Thermal dose slices were calculated from both simulated and clinical MR‐thermometry slices via the Dewey–Sapareto dose formalism using linear interpolation in time points between consecutive slices occurring 2.57 s apart to obtain a finer temporal discretization with dt=.0257s. In the patient treatments, the initial body temperature was set to a value of ear temperature of the patient measured before treatment. These temperature values for patients 1 to 7 were 37.1, 37.6, 37.2, 37.3, 36.5, 35.5, and 36.7 °C, respectively. These values were used as the initial temperature value for calculating thermal dose from the patient treatment MR‐thermometry.

### 2.6 Comparison of clinical data to simulation results

The spatial accuracy of simulated sonications was assessed by comparing the AP, right–left (RL), and foot–head (FH) coordinates of the centers of mass of the thermal dose distributions of the last simulated and last treatment thermal dose slice. The assessment of the AP coordinates was performed on the sagittal slice, while the assessment of the RL and FH coordinates was performed on the central coronal slice. The 240EM_43_ thermal dose distribution contours were calculated from both the last simulated and last clinical thermal dose slice, thus taking into consideration the MR‐thermometry slices at all of the previous time points.

To assess the accuracy of the simulations pertaining to the magnitude of temperature rise, two metrics were used: peak temperature curves as a function of time evaluated using the sagittal slice of simulation and of treatment as well as thermal dose threshold volume sizes. The 240EM_43_ focal volume thermal dose distribution sizes were compared by comparing the 240EM_43_ thermal dose focal volumes obtained via voxel summation of clinical and simulated coronal slices.

## 3 RESULTS

### 3.1 Phantom data

The depths at which sonications were performed for the phantom validation of the simulation pipeline are illustrated in Figure 1a. Results of implementing the simulation pipeline are shown in Figures 1b,c. The treatment cell position and thermal dose centers of mass of simulation and treatment all remained within about 1 voxel of each other both in the FH and RL directions. The misregistration correction values of the phantom sonications corresponding to these results are zero. This figure demonstrates that simulations are able to model the temperature rise magnitude and location of the thermal focus with good agreement in the phantom.

### 3.2 Evaluation of simulation accuracy compared to patient treatment results using three values of fibroid absorption

**FIGURE 2:**
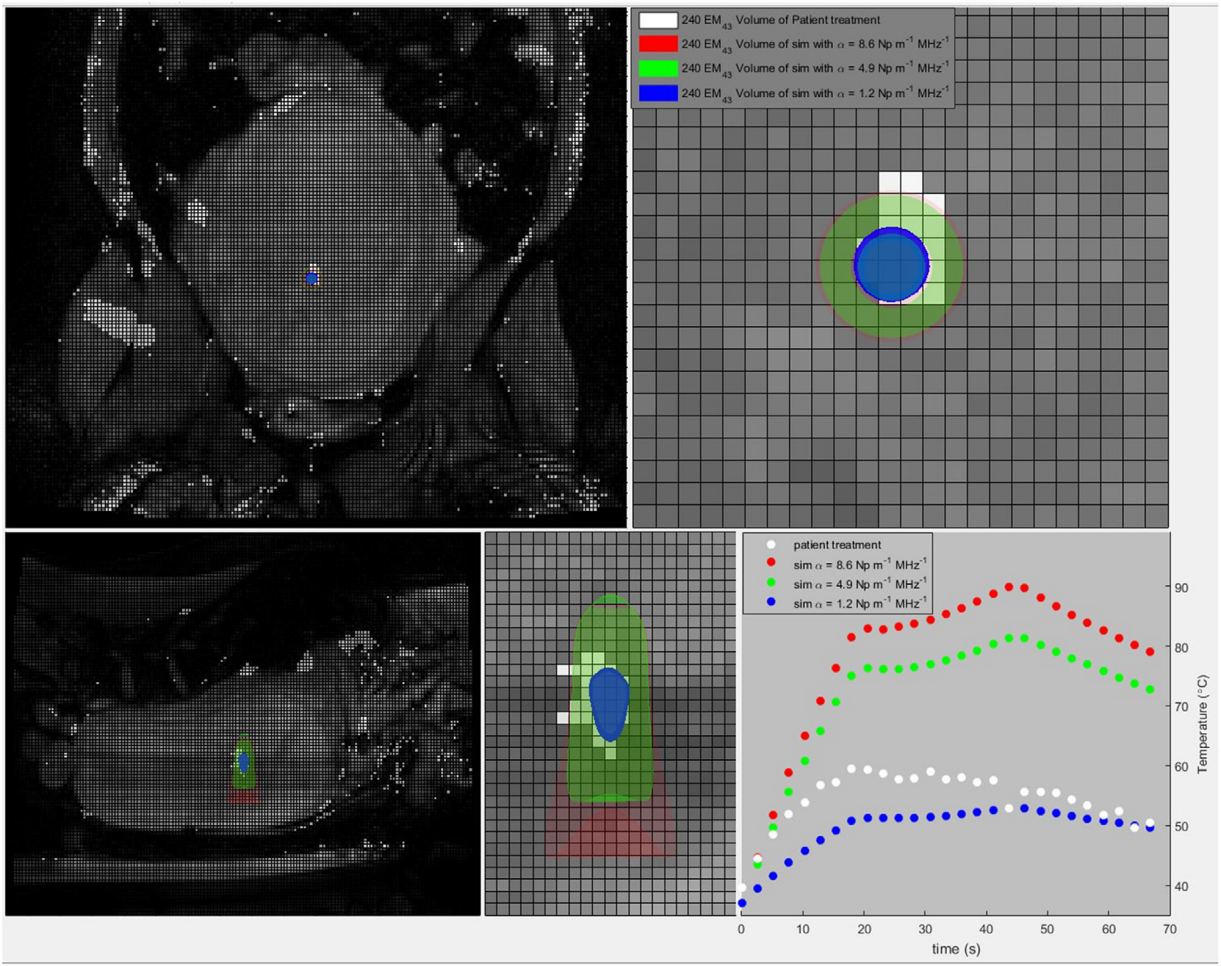
Simulated 240EM_43_ thermal dose distributions coregistered with patient anatomy and the last thermal dose slice along with the corresponding peak temperature–time curves of the sagittal slice. The blue, green, and red volumes denote simulations with values of alpha of 1.2, 4.9, and 8.6 Np m^–1^ MHz^–1^, respectively, while the white denotes the 240EM_43_ region calculated from the clinical MR‐thermometry slices. All voxels shown are 2.1 by 2.1 mm. Top‐left: posterior coronal slice showing a location of sonication with respect to patient anatomy. Top‐right: a zoomed‐in section of the coronal slice shown on the top left. Bottom‐left: sagittal slice showing the location of the sonication with respect to patient anatomy. Bottom‐middle: a zoomed‐in section of the sagittal slice. Bottom‐right: sagittal peak temperature–time curve which corresponds to the simulated and calculated thermal dose distributions shown in this figure

Simulated 240EM_43_ thermal dose distributions coregistered with patient anatomy and the last thermal dose slice along with the corresponding peak temperature–time curves of the sagittal slice are shown in Figure 2 for a sonication for which the simulation results of coronal 240EM_43_ thermal dose volume sizes and sagittal temperature curves both provide approximate bounds for the patient treatment results. This sonication utilized trajectory times of 18.8 and 26.1 s for the first and second trajectories respectively which can be seen as the changes in slopes of the temperature curve.

**FIGURE 3:**
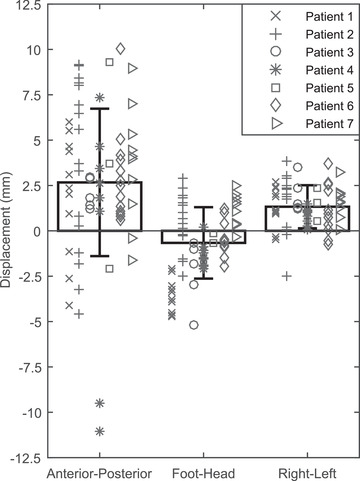
The coordinate‐wise differences between the simulated and measured thermal dose center of masses are shown for *N* = 67 sonications. For example, a positive value for an AP displacement means that the simulation resulted in a thermal dose center of mass posterior of the treatment. The values of absorption and perfusion used were 4.9 Np m^–1^ MHz^–1^ and ω = 1.89 kg m^–3^ s^–1^

The coordinate‐wise differences between the simulated and measured thermal dose center of masses are shown in Figure 3 for *N* = 67 sonications. The results show that in most cases the focus location can be accurately predicted by the simulations with the average error ± one standard deviation of 2.7±4.1 mm, –0.7±2.0 mm, and 1.3±1.2 mm in the AP, FH, and RL directions, respectively.

Ratios of sagittal MR‐thermometry peak temperature rise of simulation to patient treatment averaged over sonications within individual patient treatments as well as throughout patient treatments are shown in Table 2. These simulations utilized a perfusion value of 1.89 kg m^–3^ s^–1^. The data in the top part of the table correspond to the time of the second simulated MR‐acquisition at which time none of the sonications have yet switched trajectories from the innermost trajectory. The data in the bottom part of the table correspond to the time of the last simulated MR acquisition before the end of simulated sonication. The first timepoint provides a means for evaluating effects due to absorption as it is early in the sonication, where perfusion effects are generally speaking not substantial^53^ and the sonications are all being performed on the same (innermost) sonication trajectory. The second time point allows one to assess the practical cumulative effects on temperature rise occurring over the entire duration of the sonication also due to perfusion as well as variability over sonication trajectories and durations from sonication to sonication. The timepoint of the end of sonication generally varies from sonication to sonication (e.g., since a substantial portion of the sonications are feedback sonications). These timepoints have a mean ± SD of 23.99 ± 10.21 s and a range of 5.14–43.69 s. The timepoints refer to time after the beginning of simulated sonication (see Supporting Information for details involving temporal coregistration of MR‐thermometry dynamics).

**TABLE 2:** Top: Temperature rise ratio (mean ± SD) of simulation to treatment at the time of second simulated MR‐acquisition (5.14 s after the beginning of simulated sonication). Bottom: Temperature rise ratio (mean ± SD) of simulation to treatment at the time of last simulated MR‐acquisition before end of sonication (timepoints have mean ± SD of 23.99 ± 10.21 s and range 5.14−43.69 s)

**Absorption coefficient**	**Patient [number of sonications]**							
**(m^−1^ MHz^−1^)**	**1 [11]**	**2 [16]**	**3 [5]**	**4 [9]**	**5 [3]**	**6 [13]**	**7 [10]**	**All [67]**
8.6^a^	1.44 ± 0.39	1.29 ± 0.42	2.52 ± 0.66	1.23 ± 0.33	2.32 ± 0.49	1.18 ± 0.38	1.16 ± 0.26	1.40 ± 0.55
4.9^b^	1.14 ± 0.30	1.17 ± 0.37	2.15 ± 0.59	1.08 ± 0.30	2.04 ± 0.43	1.06 ± 0.40	1.00 ± 0.22	1.22 ± 0.48
1.2^c^	0.39 ± 0.10	0.46 ± 0.14	0.79 ± 0.23	0.41 ± 0.11	0.77 ± 0.16	0.42 ± 0.19	0.37 ± 0.08	0.46 ± 0.19
**(m^−1^ MHz^−1^)**	**1 [11]**	**2 [16]**	**3 [4]**	**4 [9]**	**5 [3]**	**6 [13]**	**7 [10]**	**All [66]**
8.6	1.56 ± 0.36	1.38 ± 0.49	3.73 ± 1.10	1.38 ± 0.39	1.46 ± 0.14	1.22 ± 0.20	1.15 ± 0.20	1.49 ± 0.71
4.9	1.23 ± 0.27	1.24 ± 0.40	3.18 ± 0.93	1.21 ± 0.35	1.25 ± 0.11	1.08 ± 0.21	0.97 ± 0.15	1.28 ± 0.60
1.2	0.42 ± 0.09	0.47 ± 0.15	1.16 ± 0.33	0.45 ± 0.13	0.45 ± 0.04	0.41 ± 0.11	0.35 ± 0.05	0.47 ± 0.22

^a^ Keshavarzi et al.,^28^, Liu et al.,^32^, Ellens and Hynynen.^33^

^b^ Average of 1.2 and 8.6 m^−1^ MHz^−1^.

^c^ Siddiqi et al.^52^

**FIGURE 4:**
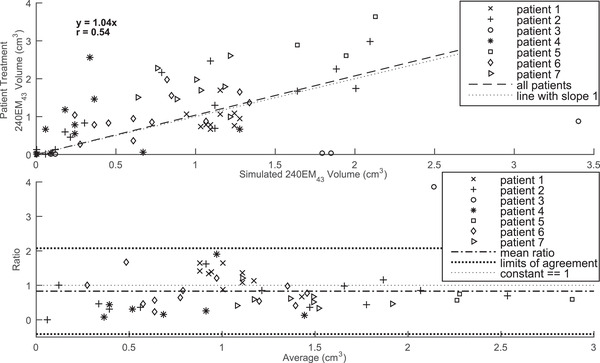
Top: a linear regression and scatter plot of the 240EM_43_ thermal dose volumes of 67 sonications of patient treatments and simulations utilizing a fibroid absorption value of 4.9 Np m^–1^ MHz^–1^. Bottom: a Bland–Altman plot with ratios of 240EM_43_ volumes of simulations to treatment as a function of the average of the two quantities of *N* = 54 sonications each having a patient treatment 240EM_43_ volume greater than 2 voxels. The limits of agreement are mean ratio ± 2 standard deviations

A linear regression and scatter plot of the 240EM_43_ thermal dose volumes of 67 sonications of patient treatments and simulations utilizing a fibroid absorption value of 4.9 Np m^–1^ MHz^–1^ are shown in the top panel of Figure 4. The slope of the regression line is 1.04 and the correlation coefficient is 0.54. The corresponding linear regression analysis on the simulation data where a fibroid absorption value of 8.6 Np m^–1^ MHz^–1^ was used yielded a slope of 0.77 and a correlation coefficient of 0.56. The 240EM_43_ volumes of simulations utilizing a fibroid absorption value of 1.2 Np m^–1^ MHz^–1^ and a perfusion value of 1.89 kg m^–3^ s^–1^ were zero for 60 of the 67 sonications so neither regression nor Bland–Altman analyses are shown for this value of fibroid absorption (see Supporting Information). Regression slopes and correlation coefficients of thermal dose volumes of patient treatment versus simulation for various other threshold values used in the literature^3^, ^24^, ^33^ can be found in Table 3.

**TABLE 3:** Regression slopes and correlation coefficients of thermal dose volumes of patient treatment vs. simulation for various threshold values

**Absorption coefficient (m^−1^ MHz^−1^)**	**Thermal dose volume threshold value (EM_43_)**	**Regression slope**	**Pearson correlation coefficient**
4.9	5	1.29	0.79
4.9	18	1.24	0.73
4.9	30	1.21	0.70
4.9	240	0.77	0.56
8.6	5	1.04	0.79
8.6	18	0.97	0.74
8.6	30	0.93	0.72
8.6	240	1.04	0.54

A Bland–Altman plot with ratios of 240EM_43_ volumes of simulations utilizing a fibroid absorption value of 4.9 Np m^–1^ MHz^–1^ to treatment of *N* = 54 sonications each having a patient treatment 240EM_43_ volume greater than 2 voxels is shown in the bottom panel of Figure 4. The dashed lines in the plot represent the limits of agreement, mean ratio ± 2 standard deviations of the ratios of simulation to treatment 240EM_43_ thermal dose volumes. The values of the mean ratio, lower and upper limits of agreement are 0.83, –0.41, and 2.08 respectively for the simulations utilizing a fibroid absorption value of 4.9 Np m^–1^ MHz^–1^. Performing the corresponding Bland–Altman analysis on the simulations utilizing a fibroid absorption coefficient of 8.6 Np m^–1^ MHz^–1^ yielded mean ratio, lower and upper limits of agreement values of 1.20, –0.45, and 2.84, respectively. Of the 67 sonications simulated and compared to patient treatment, the 240EM_43_ patient treatment volumes in voxels were zero for eight sonications, one for three sonications, and two for two sonications, leaving *N* = 54 sonications having a patient treatment volume greater than 2 voxels. Five of the eight sonications having patient treatment 240EM_43_ volumes of 0 voxels had simulated 240EM_43_ volumes of 0 voxels when utilizing fibroid absorption values of 4.9 and 8.6 Np m^–1^ MHz^–1^, leaving three sonications with simulated 240EM_43_ volumes greater than zero voxels when utilizing either of the aforementioned absorption values.

### 3.3 Variation of perfusion

The effect of perfusion on center of mass (of thermal dose distribution) was assessed. When comparing the AP location of the center of mass of a thermal dose distribution of the sagittal slice of a sonication simulated using a value of perfusion of 1.89 kg m^–3^ s^–1^ to that of a higher value, the results demonstrated that perfusion changes had only a small impact on the thermal dose location (see Figure S2 and text in Supporting Information for details). For example, the effect sizes of varying perfusion for simulations utilizing a fibroid absorption value of 8.6 Np m^–1^ MHz^–1^ on changes in displacements remained under 1 voxel. The analogous comparison for the FH and RL displacements yielded values less than 1 mm in all simulations in which perfusion was varied.

**FIGURE 5:**
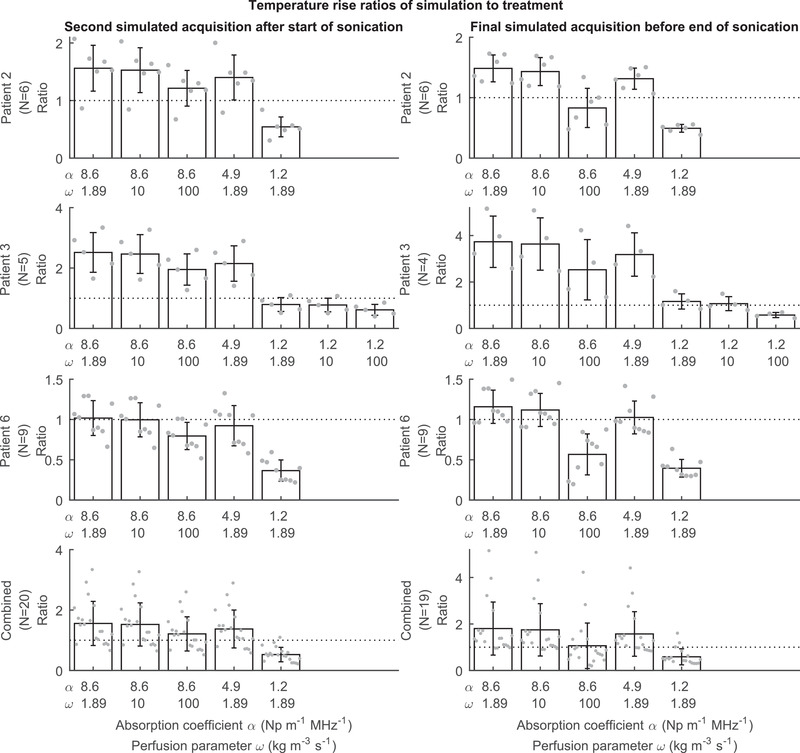
Effects of varying perfusion and absorption on temperature rise are shown for *N* = 20 sonications by using ratios of sagittal MR‐thermometry peak temperature rise of simulation to patient treatment averaged over sonications within individual patient treatments as well as throughout patient treatments. The averaging was performed at the time of the second simulated MR‐acquisition which is 5.14 s after the start of simulated sonication (left column) and at the time of the last simulated MR‐acquisition before the end of sonication (right column)

Effects of varying perfusion and absorption on temperature rise are shown in Figure 5 for *N* = 20 sonications using the same timepoints that were used in Table 2. The results of individual patients demonstrate that fibroid absorption appears to vary from patient to patient, while perfusion alone cannot explain the degree of low clinical heating exhibited by patient 3: First, patient 3 shows the best agreement with a fibroid absorption value of 1.2 Np m^–1^ MHz^–1^ unlike patients 2 and 6, where results show better agreement with higher values of fibroid absorption. Second, varying perfusion while using a higher value of fibroid absorption in patient 3 is unlikely to bring the results into a good agreement. In the two sonications of patient 2 simulated using absorption value of 1.2 Np m^–1^ MHz^–1^ in which perfusion was varied, changing perfusion from 1.89  to 10 kg m^–3^ s^–1^ resulted in simulated max temperature differences during heating of at most 1.23 °C.

**FIGURE 6:**
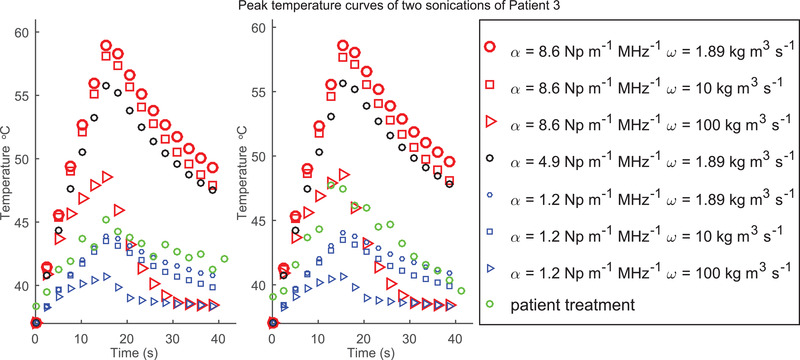
Peak temperature–time curves of the sagittal MR‐thermometry slice of simulations utilizing different combinations of fibroid perfusion and absorption parameters as well as those of the patient treatment are shown for two sonications of patient 3

Peak temperature–time curves of the sagittal MR‐thermometry slice of simulations utilizing different combinations of fibroid perfusion and absorption parameters as well as those of the patient treatment are shown in Figure 6 for two sonications of patient 3. This figure provides an example of a sonication showing agreement with a fibroid absorption value of 1.2 Np m^–1^ MHz^–1^ (left) as well as an example illustrating that atypically high values of perfusion may be an important factor contributing to clinical heating (right). These data show that perfusion can have an effect on peak temperature curves.

The ratio of the 240EM_43_ thermal dose volume of a sonication obtained via simulation by utilizing a perfusion parameter of 10 kg m^−3^ s^−1^ to that of 1.89 kg m^−3^ s^−1^ ranged from 0.61 to 1.00 for *N* = 19 of the sonications simulated utilizing an absorption value of 8.6 Np m^−1^ MHz^−1^ (see Figure S8 for details).

### 3.4 Variation of absorption

**FIGURE 7:**
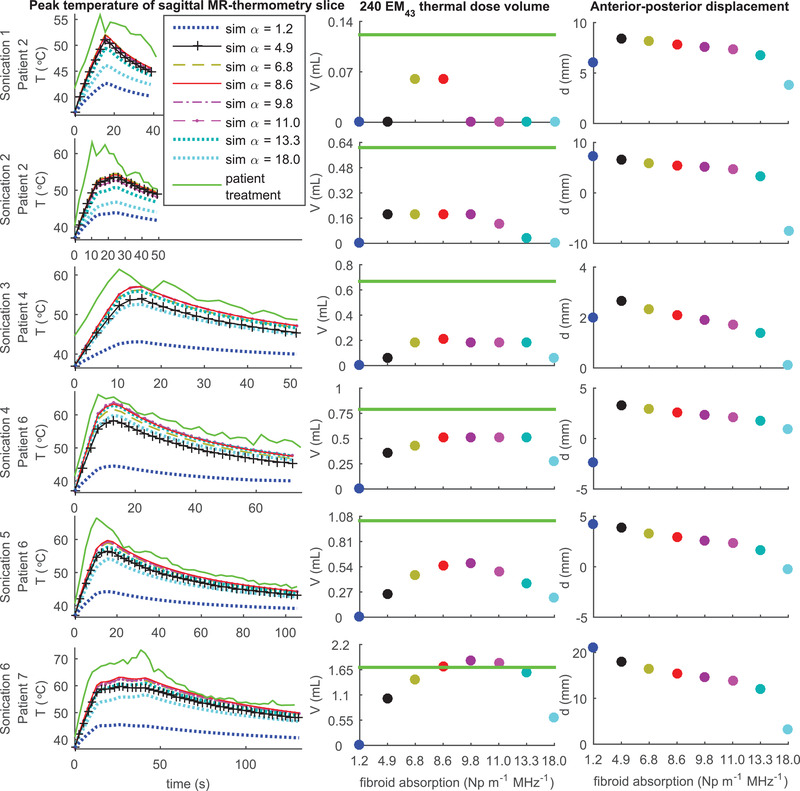
Peak temperature curves (column 1) and 240EM_43_ thermal dose threshold volumes (column 2) of simulations and treatment along with displacements (column 3) between centers of mass of thermal dose distribution of simulation and treatment of six sonications for which a fibroid absorption value of 8.6 Np m^–1^ MHz^–1^ did not show a high degree of heating when compared to patient treatment results are shown for multiple values of fibroid absorption. In the displacement plots, increasing *d* is in the posterior direction. Furthermore, a positive value of AP displacement means that the simulation resulted in a thermal dose center of mass posterior of the treatment

Peak temperature curves and 240EM_43_ thermal dose threshold volumes of simulations and treatment as well as displacements between the centers of mass of the thermal dose distribution of the sagittal MR‐thermometry slice of simulations and treatment in the AP direction are shown as a function of fibroid absorption for six sonications in Figure 7. As fibroid absorption was varied from 1.2 to 18.0 Np m^–1^ MHz^–1^, for each sonication these displacements varied by less than 0.5 mm in the RL direction and by less than 0.6 mm in the FH direction (see Figure S3 for details). The temperature curves and 240EM_43_ volumes demonstrate that the absorption parameter value of 8.6 Np m^–1^ MHz^–1^ appears to produce a degree of heating that is nearly maximal for all of the sonications. The simulations not showing a high degree of heating when compared to patient treatment results in these sonications thus could not be explained by homogenously varying absorption (along with attenuation) within physiologically reasonable parameter ranges throughout the entire fibroid while keeping other parameters at their particular values. It is unlikely that simulations of these sonications not showing a high degree of heating compared to treatment results could be explained by perfusion lower than 1.89 kg m^–3^ s^–1^ as perfusion effects of parameters within such a range are not substantial during intervals of the order of 10–20 s.^53^ The individual sonications illustrate that the acoustic field at a particular location computed using this particular model as a function of the parameter alpha is not monotone increasing but concave down with a relative maximum dependent of location which is due to using the same parameter value for attenuation and absorption. The displacement data show that the location of the thermal dose center of mass is not strongly dependent on absorption parameter at least for values between 4.9 and 13.3 Np m^–1^ MHz^–1^ but rather suggests that a posterior shift of the simulated acoustic focus (due to near‐field acoustic parameter related focusing effects) may have contributed to the shift as well as the lack of a high degree of simulated heating when compared to patient treatments. For example, if the differences of the characteristic acoustic impedances between adjacent near‐field tissues that sound is propagating through would cause a posterior shift of the focus, one would also expect the focus to be larger in volume which could lead to less localized heating.

## 4 DISCUSSION

This is the first study comparing numeric simulations with actual clinical MRI‐guided FUS treatments of UFs. The results show that the focal location can be accurately predicted and that the accuracy of the temperature elevation prediction varies from location to location in a patient and from patient to patient. This agrees with the earlier reports showing that the temperature elevation induced in fibroids was highly variable.^3^ The simulations indicate that these variations are due to local tissue absorption of ultrasound and to a smaller degree on the blood perfusion in the targeted tissue location. By adjusting these parameters, the measured temperature curves could be reproduced.

The degree of spatial accuracy of simulations means that the simulations show promise in applications of treatment planning as simulations are accurate to within several voxels for the majority of cases. In most cases, displacements in the AP direction were less than 3 voxels (for alpha 4.9 and 8.6 Np m^–1^ MHz^–1^); and those in the RL and FH directions were less than 2 voxels (Figure 3). The aforementioned displacements are similar in magnitude to the displacements presented by Kim et al.^18^ between the thermal dose center of mass of patient treatment and sonication target. This ability to predict the location of the hotspot did not strongly depend on perfusion or ultrasound absorption which were dominating in determining the temperature elevation.

The ability to predict temperature elevation was reasonable on average, but there were large variations from sonication to sonication when fixed tissue parameters were used. The large variation between sonications is consistent with earlier observations by McDannold et al.^3^ However, a good match of individual sonications could be obtained by tuning the local ultrasound absorption coefficient of the fibroid. For example, sonications of the treatment of patient 3 exhibited a low degree of clinical heating. A good agreement between the peak temperature curves of simulations and measurement was found by utilizing a value of fibroid absorption of 1.2 Np m^–1^ MHz^–1^ (Table 2, Figure 5). We also explored the question of whether or not it is possible that all of the fibroids have nearly the same absorption and tuning the perfusion rate could be used to achieve a good agreement. The results suggest that while atypically high values of perfusion may be an important factor contributing to low clinical heating, perfusion alone cannot explain the low heating exhibited clinically by patient 3 and that low absorption has an important role. The aforementioned agreement between the simulation data utilizing a perfusion parameter of 1.89 kg m^–3^ s^–1^ and a value of absorption of 1.2 Np m^–1^ MHz^–1^ suggests that there may be UFs having values of fibroid absorption as low as 1.2 Np m^–1^ MHz^–1^. These results (as well as Figure S4) suggest that absorption is an important tissue parameter to take into account when predicting temperature rise for patient‐specific sonication strategies. The parameter values of uterus/fibroid attenuation utilized in this study are comparable to parameter values available in the literature in the context of in vivo human studies.^30^, ^31^, ^52^

While the peak temperature curves of simulations utilizing values of fibroid absorption of 4.9 and 8.6 Np m^–1^ MHz^–1^ yielded results that were in good agreement with the treatment results of patient 4 for most of the sonications, two of the sonications which were performed on the same treatment cell position located on or close to the border/septum of the fibroid showed considerable overheating in simulations compared to that of treatments of about a factor of 1.5 or more. Visual inspection of the sagittal MR‐thermometry slices of the patient treatment as a function of time of one of these sonications revealed a region of cooling shaped like a vessel within the region of the focal volume heating, suggesting that cooling due to the presence of a thermally significant blood vessel not accounted for within the simulations had a contribution to the discrepancy between magnitudes of heating exhibited in simulations and treatments (Figures S6 and S7). This is consistent with earlier simulation^54^ and experimental^55^ studies. The data also suggest that this kind of vessel‐like structure seen on the MR‐thermometry can cause an exaggerated thermal dose center of mass displacement between simulations and treatment in the anterior direction, as the two outlier data points of AP displacements of *N* = 67 sonications of simulations correspond to the two sonications performed at the location showing this vessel‐like structure (Figure 3).

The 240EM_43_ thermal dose threshold volumes of simulations utilizing values of alpha of 4.9 and 8.6 Np m^–1^ MHz^–1^ showed a moderate degree of correlation to those of the patient treatments. The mean ratios of simulation to treatment dose volume being within about 20% and thus showing good agreement on average, but the degree of spread in the ratios was large (Figure 4). The regression line of the simulations utilizing the value of fibroid absorption of 4.9 Np m^–1^ MHz^–1^ shows overall agreement with a slight degree of underprediction by having a slope close to but slightly over one while that of the simulations utilizing a value of fibroid absorption of 8.6 Np m^–1^ MHz^–1^ indicates overall overprediction.

The likely reason for the high degree of spread in the ratios of the 240EM_43_ thermal dose threshold volumes of simulations to those of treatments (e.g., Figure 4) is that tissue parameters of absorption, perfusion, and thermal conductivity vary as was discussed above. Some literature also supports that this may be the case, e.g., while one may consider 10 kg m^–3^ s^–1^ to be an upper bound for tumors commonly found in humans,^56^ the Pennes perfusion parameter of UFs was estimated to have a mean ± SD of 11.0 ± 11.6 kg m^–3^ s^–1^ with an approximate range of 0 to 30 kg m^–3^ s^–1^ by Dillon et al.^25^ Estimates of thermal conductivity of UFs available in the literature indicate that thermal conductivity of UFs appears to vary. Thermal diffusivity of UFs was estimated using the heating data of nine low power sonications in eight UF patients by Dillon et al.^25^ which, when converted to thermal conductivity values using the heat capacity and density values of Table 1 yields values of κ = 0.77 ± 0.41 W m^–1^ K^–1^ (mean ± SD) with an approximate range of 0.41 to 1.49 W m^–1^ K^–1^. Thermal conductivity of UFs has also been estimated by Zhang et al.,^39^ by using the cooling data from 81 high power (80–200 W) sonications in nine UF patients, to have a median ± SD of 0.47 ± 0.07 W m^–1^ K^–1^ with a range of 0.25–0.67 W m^–1^ K^–1^. While the value of thermal conductivity of Table 1 was within these ranges reported in the literature and may be close on average to that of the patients, the aforementioned literature values suggest that variation of the thermal conductivity may have contributed to the spread in the ratios of the 240EM_43_ thermal dose threshold volumes of simulations to those of treatments. All of these tissue properties may vary within a single fibroid.

While the simulations indicate that the variations in peak temperature elevation are due to local tissue absorption of ultrasound and to a smaller degree on the blood perfusion in the targeted tissue location, the effect on 240EM_43_ thermal dose threshold volumes due to varying perfusion can still be substantial (Figure S8) suggesting that perfusion is also a clinically significant tissue parameter. Furthermore, quantitative perfusion estimates obtained via dynamic contrast‐enhanced MRI of one patient exhibiting low clinical heating, obtained in a study by Suomi et al.,^26^ suggest that perfusion may be an important factor in clinical heating. In addition, practically from a medical perspective, it is also possible that absorption and perfusion are related in the sense that if one were to administer a vasoactive drug such as leuprolide acetate in an effort to reduce perfusion within UFs,^57^ then the biophysical properties of the tissue could change and manifest as an increase in ultrasound absorption. A difference between RF‐heating and MRgFUS is that the ultrasound exposures typically are shorter and thus not dominated by perfusion. Perfusion still has an impact on temperature elevation but not as large as during longer exposures used typically in RF‐heating. It is simply proportional to the volume of blood that is heated when compared with the heated tissue volume. The longer the heating duration the larger the blood volume flowing through the heated tissue and the larger its impact on the temperature elevation. For example, in one study, Milic et al.^58^ when performing radiofrequency ablation of UFs, the authors reported a mean heating cycle duration of 4.4 min which is much longer than the sonications included in this study (see Table 2).

Unknown tissue‐specific properties, which provide an explanation to low heating as well as off‐target heating at least in some cases, present a challenge that remains to be addressed. Considering that the model utilizing constant tissue parameters yielded reasonable agreement with temperature rise and lesion size on average but was not able to predict these accurately for individual sonications indicates that with simulations it may be possible to further improve proposed clinical treatment strategies^59^, ^60^ by assessing performance on average^33^ but currently cannot be used to optimize sonication specific parameters during treatments due to lack of a priori knowledge of tissue parameters. In addition, exploring further the effects of varying parameters in the tissue parameter space on simulated treatments as well as performing a comparison of thermal dose distributions resulting from simulations of full treatments (all sonications) to those measured by MR‐thermometry as well as to nonperfused volumes could form an area of future work. However, performing an in vivo validation of this simulation model within UF patient geometry involving the near‐field fat and far‐field bone is difficult due to the challenge of monitoring temperature distributions reliably in fat^61^ and bone.^62^

Limitations of this study include a small number of patients and thermometry accuracy. Limitations of MR‐thermometry accuracy include assuming a value of −0.0094 ppm/°C for the temperature sensitivity coefficient (constant of proportionality between phase and temperature), which being difficult to determine precisely in vivo exhibits variation between and within biological tissues^62^; properties of lipids described in Stafford and Hazle^63^; as well as contributions to uncertainty from noise, small motion artifacts, and volume‐averaging effects.^3^ Considering that coronal slice thickness is 7 mm, the partial volume effects may contribute to error in the 240EM_43_ volumes. Neither the phantom simulation nor the phantom experiment had perfusion and thus serve only as a partial validation of the simulation model. Furthermore, as discussed in Mahoney et al.^34^ the Pennes bioheat equation only approximates the effects of perfusion. The perfusion value of 100 kg m^−3^ s^−1^ was selected to represent an atypically high value of perfusion in an effort to assess potential effects of varying UF perfusion. However, as the literature estimates of quantitative fibroid perfusion parameters^25^, ^26^ are limited to a feasibility study demonstrating an estimation technique and a case study of one patient involving low clinical heating, and considering that in studies involving larger patient sizes^20^, ^21^ some semiquantitative perfusion parameters varied over a range between one and two orders of magnitude, the least upper bound of physiological perfusion values of UFs is difficult to estimate and thus the value of 100 might be larger than physiological values. Simulation time for a sonication depends on multiple factors. For the acoustic simulations, one factor is the size of the contours in which velocity is calculated, the choice of which is influenced by the depth of sonication as well as patient geometry (in order to have transducer element surface normal vectors intersect with contours of the layered model). Another factor is the number of trajectories on a treatment cell. Thermal simulations depend on the number of timesteps which depends on the durations of the trajectories as well as the cooling interval. Other limitations (see Supporting Information) are that the layered model serves only as an approximation of the patient anatomy, and that several approximations involving the treatment device were made in the implementation of the simulations including uniform velocity of transducer elements and use of nominal power values in log files.

## 5 CONCLUSIONS

As a conclusion, the results of this study suggest that perfusion, while in some cases having a substantial impact on thermal dose volumes, has less impact than ultrasound absorption for predicting peak temperature elevation at least for this particular array geometry, frequencies, and tissue target which is good for clinicians to be aware of. While the results of this study suggest that these simulations show promise in applications for model‐based treatment planning of UF treatments they also indicate that the temperature elevation or lesion size induced by individual sonications is not accurately predicted by the model. While the accuracy of the model is highly dependent on accurate estimates of acoustic absorption and perfusion, lesion size is underpredicted on average by roughly 20% when using values of 4.9 Np m^−1^ MHz^−1^ and 1.89 kg m^−3^ s^−1^. This indicates the need for online temperature or tissue coagulation monitoring to assure adequate treatment effects. The simulations could be approved if the local tissue properties especially the local ultrasound absorption coefficient was known. Therefore, it may be possible to utilize model‐based treatments in the future if tissue parameters can be mapped prior to the treatment with adequate spatial resolution. Currently, such maps are not available and thus simulations can only be used for planning purposes indicating sonication performance on average.

## CONFLICT OF INTEREST STATEMENT

Dr. Elizabeth David is a principal investigator for Arrayus HIFU evaluation on fibroids. The authors don't have any other relevant conflicts of interest to disclose.

## Supporting information



SUPPORTING INFORMATION

## Data Availability

The clinical research data is not shared.
