# Supplementary material for: Comparison of computer simulations and clinical treatment results of magnetic resonance‐guided focused ultrasound surgery (MRgFUS) of uterine fibroids
Source: Med Phys. 2022 Mar 2;49(4):2101–19. doi: 10.1002/mp.15263 (PMC9314069; doi:10.1002/mp.15263)
Supplement: Supplementary file 1 — SUPPORTING INFORMATION [file MP-49-2101-s001.pdf]

Supplementary Section

Table S1: Parameters of the 67 sonications of 7 patient treatments selected for simulations.

index	Patient	Power (W)	Frequency (MHz)	Location: Treatment cell position coordinates (m)			Treatment cell diameter (mm)	Trajectory durations (s)				misregistration correction (m)		
				Anterior-Posterior	Right-Left	Foot-Head		1	2	3	4	Anterior-Posterior	Right-Left	Foot-Head
1	1	80	1.2000	0.0028	-0.0184	0.0072	4	15.90	0.00	0.00	0.00	0.0000	0.0000	0.0000
2	1	240	1.2000	0.0028	-0.0184	0.0072	4	16.00	0.00	0.00	0.00	-0.0062	0.0014	-0.0044
3	1	240	1.2000	0.0028	-0.0201	0.0122	4	16.00	0.00	0.00	0.00	-0.0062	0.0014	-0.0044
4	1	260	1.2000	0.0028	-0.0062	0.0182	4	14.20	0.00	0.00	0.00	-0.0062	0.0014	-0.0044
5	1	220	1.2000	0.0028	-0.008	0.0072	8	7.60	12.40	0.00	0.00	-0.0062	0.0014	-0.0044
6	1	220	1.2000	0.0028	0.0059	0.0072	8	7.60	12.40	0.00	0.00	-0.0062	0.0014	-0.0044
7	1	240	1.2000	0.0028	0.0163	0.0052	4	16.00	0.00	0.00	0.00	-0.0062	0.0014	-0.0044
8	1	240	1.2000	0.0028	0.018	-0.0078	4	16.00	0.00	0.00	0.00	-0.0062	0.0014	-0.0044
9	1	260	1.2000	0.0028	0.018	-0.0158	4	16.00	0.00	0.00	0.00	-0.0062	0.0014	-0.0044
10	1	260	1.2000	0.0028	0.018	-0.0018	4	16.00	0.00	0.00	0.00	-0.0062	0.0014	-0.0044
11	1	230	1.2000	0.0028	0.0042	-0.0138	8	7.60	12.40	0.00	0.00	-0.0062	0.0014	-0.0044
12	2	40	1.2000	0.0264	-0.0048	-0.0206	12	16.00	0.00	0.00	0.00	0.0000	0.0000	0.0000
13	2	210	1.2000	0.0115	0.0018	-0.0134	12	22.68	0.00	0.00	0.00	-0.0056	0.0012	0.0004
14	2	220	1.2000	0.0264	-0.0048	-0.0206	12	16.69	14.55	11.13	0.00	-0.0005	0.0009	-0.0012
15	2	100	1.2000	0.0115	0.0087	-0.0134	14	16.00	0.00	0.00	0.00	0.0000	0.0000	0.0000
16	2	220	1.2000	0.0115	0.0087	-0.0134	14	6.85	0.00	0.00	0.00	-0.0056	0.0012	0.0004
17	2	220	1.2000	0.0115	0.007	-0.0144	12	11.13	10.27	3.00	0.00	-0.0056	0.0012	0.0004
18	2	200	1.2000	0.0115	-0.0138	-0.0264	12	20.97	7.70	7.70	0.00	-0.0056	0.0012	0.0004
19	2	210	1.2000	0.0115	0.0018	-0.0294	12	13.27	5.14	15.41	0.00	-0.0056	0.0012	0.0004
20	2	180	1.2000	0.0115	-0.0121	-0.0074	12	10.70	7.70	15.41	0.00	-0.0056	0.0012	0.0004
21	2	180	1.2000	0.0115	-0.0017	-0.0054	12	13.27	2.57	23.11	0.00	-0.0056	0.0012	0.0004
22	2	100	1.4443	0.0035	-0.0081	0.0106	8	16.00	0.00	0.00	0.00	0.0000	0.0000	0.0000
23	2	150	1.4443	0.0035	-0.0219	0.0046	8	13.70	10.70	0.00	0.00	0.0046	0.0023	-0.0017
24	2	140	1.4443	0.0035	0.004	0.0016	8	26.53	2.57	0.00	0.00	0.0046	0.0023	-0.0017
25	2	180	1.2000	0.0115	0.007	-0.0224	12	13.70	10.70	19.26	0.00	-0.0056	0.0012	0.0004
26	2	190	1.2000	0.0115	-0.0294	0.0026	8	11.56	0.00	0.00	0.00	-0.0056	0.0012	0.0004
27	2	150	1.4443	0.0035	0.0162	-0.0194	8	16.69	7.70	0.00	0.00	0.0046	0.0023	-0.0017
28	3	100	1.2000	-0.007	0.0209	-0.0237	12	16.00	0.00	0.00	0.00	0.0000	0.0000	0.0000
29	3	200	1.2000	-0.007	0.0209	-0.0237	12	27.82	0.00	0.00	0.00	0.0000	0.0018	-0.0018
30	3	210	1.2000	-0.007	0.0209	-0.0337	12	18.83	26.11	0.00	0.00	0.0000	0.0018	-0.0018
31	3	100	1.4443	-0.0133	0.0289	-0.0267	12	16.00	0.00	0.00	0.00	0.0000	0.0000	0.0000
32	3	190	1.4443	-0.0133	0.0289	-0.0267	12	26.53	0.00	0.00	0.00	-0.0033	0.0011	-0.0016
33	4	160	1.2000	0.0076	0.0019	0.0213	12	16.26	1.28	0.00	0.00	-0.0016	0.0019	-0.0014
34	4	160	1.2000	0.0076	0.0019	0.0213	12	13.70	0.00	0.00	0.00	-0.0016	0.0019	-0.0014
35	4	160	1.2000	0.0076	-0.0068	0.0163	12	14.12	7.28	15.41	0.00	-0.0016	0.0019	-0.0014
36	4	160	1.2000	0.0076	-0.0155	0.0133	12	25.68	0.00	0.00	0.00	-0.0016	0.0019	-0.0014
37	4	160	1.2000	0.0076	-0.0155	0.0133	12	37.23	0.00	0.00	0.00	-0.0016	0.0019	-0.0014
38	4	170	1.2000	0.006	-0.0206	0.0295	8	13.70	10.27	0.00	0.00	-0.0016	0.0019	-0.0014
39	4	170	1.2000	0.006	-0.0051	0.0305	8	13.70	2.57	0.00	0.00	-0.0016	0.0019	-0.0014
40	4	170	1.2000	0.006	0.0071	0.0235	8	13.27	3.00	0.00	0.00	-0.0016	0.0019	-0.0014
41	4	170	1.2000	0.006	-0.0137	0.0355	8	11.13	3.85	0.00	0.00	-0.0016	0.0019	-0.0014
42	5	150	1.2000	-0.0056	0.003	-0.0079	16	13.70	5.56	20.54	1.28	-0.0034	0.0014	-0.0015
43	5	180	1.2000	-0.0056	-0.0091	-0.0089	12	13.27	7.70	11.13	0.00	-0.0034	0.0014	-0.0015
44	5	180	1.2000	-0.0056	-0.0039	-0.0199	14	11.13	2.57	15.41	9.42	-0.0034	0.0014	-0.0015
45	6	180	1.2000	0.0191	-0.0237	0.0194	8	27.82	0.00	0.00	0.00	0.0007	0.0010	0.0013
46	6	180	1.2000	0.0025	-0.0168	0.0126	8	13.70	10.27	0.00	0.00	-0.0032	0.0020	0.0007
47	6	50	1.2000	-0.0058	-0.0237	0.0394	8	16.00	0.00	0.00	0.00	0.0000	0.0000	0.0000
48	6	180	1.2000	-0.0058	-0.0237	0.0394	8	11.13	7.70	0.00	0.00	-0.0036	0.0003	-0.0012
49	6	180	1.2000	0.0191	-0.0029	0.0114	12	21.40	10.70	3.00	0.00	0.0007	0.0010	0.0013
50	6	180	1.2000	0.0191	-0.0133	0.0054	12	18.40	12.84	6.85	0.00	0.0007	0.0010	0.0013
51	6	180	1.2000	0.0191	-0.0133	0.0154	12	31.67	9.84	0.00	0.00	0.0007	0.0010	0.0013
52	6	180	1.2000	-0.0058	-0.0168	0.0394	8	11.13	15.41	0.00	0.00	-0.0036	0.0003	-0.0012
53	6	180	1.2000	-0.0058	-0.0324	0.0384	8	8.56	5.14	0.00	0.00	-0.0036	0.0003	-0.0012
54	6	180	1.2000	-0.0058	-0.041	0.0394	8	8.56	7.70	0.00	0.00	-0.0036	0.0003	-0.0012
55	6	180	1.2000	-0.0058	-0.0203	0.0474	8	8.56	5.99	0.00	0.00	-0.0036	0.0003	-0.0012
56	6	180	1.2000	0.0008	-0.0376	0.0157	8	11.13	5.14	0.00	0.00	0.0018	0.0018	0.0002
57	6	180	1.2000	0.0008	-0.0307	0.0117	8	14.12	7.70	0.00	0.00	0.0018	0.0018	0.0002
58	7	50	1.2000	0.0033	0.0138	0.0105	12	16.00	0.00	0.00	0.00	0.0000	0.0000	0.0000
59	7	150	1.2000	0.0033	0.0138	0.0105	12	16.26	10.27	9.84	0.00	-0.0056	0.0009	0.0005
60	7	70	1.2000	0.0071	0.0141	0.022	12	16.00	0.00	0.00	0.00	0.0000	0.0000	0.0000
61	7	180	1.2000	0.0071	0.0019	0.015	12	13.69	12.84	14.98	0.00	0.0001	0.0011	0.0007
62	7	180	1.2000	0.0071	0.0019	0.005	12	14.12	17.97	11.56	0.00	0.0001	0.0011	0.0007
63	7	180	1.2000	0.0033	0.0034	0.0265	12	13.69	7.70	10.27	0.00	-0.0056	0.0009	0.0005
64	7	180	1.2000	0.0071	-0.0102	0.01	12	13.69	10.27	10.70	0.00	0.0001	0.0011	0.0007
65	7	180	1.2000	0.0071	-0.0067	0.022	12	14.12	7.70	8.56	0.00	0.0001	0.0011	0.0007
66	7	180	1.2000	0.0071	0.0141	0.022	12	18.83	17.97	5.99	0.00	0.0001	0.0011	0.0007
67	7	180	1.2000	0.0071	0.0245	0.028	12	21.40	7.70	2.57	0.00	0.0001	0.0011	0.0007

Note: The anterior-posterior coordinate of the treatment cell position of sonication 12 and 14 indicated in this table and used in the simulations may have been due to error 1cm posterior of the location indicated by the treatement planning system.

Table S 1 Note: The anterior-posterior coordinates of the treatment cell positions in the main body of the manuscript corresponding to depth were obtained from those of table S1 by adding 72.5 mm as the treatment planning system had a parameter denoting the top level of the liquid housing the transducer having value of 72.5 mm anterior of the isocenter of the magnet.

Implementation details of a simulated sonication

Calculation of absorbed power density

The surface velocities on the transducer elements were initially set to have magnitude 1, and the phases of the velocities on the transducer were calculated using a speed of sound value of 1540 m/s found in the treatment planning system configuration file, by back propagating from a target point calculated by using treatment parameters of the treatment planning system log and configuration files.

To take into account the acoustic power used to drive the transducer, the resulting absorbed power density Q fields were scaled based on the nominal power value in the treatment planning system log files in such a way that the power emitted by the transducer surface calculated via $\frac{1}{2}\rho_{oil}c_{oil}|u_0|^2 A_{Transducer}$ matched the nominal power value in treatment planning system log files which is assumed to be approximately equal to brush target measurements i.e. the absorbed power density fields calculated using a velocity of 1 were multiplied by $|u_0|^2 = \frac{P_{nominal}}{\frac{1}{2}\rho_{oil}c_{oil}A_{Transducer}}$. It was assumed that the power calibration of the transducer was performed while the transducer was immersed in oil, and thus the attenuation of the oil was modeled as zero in the acoustic simulations.

The sizes of the areas of the contours on which the velocity was calculated were chosen to be large enough that the surface normal vectors of the transducer elements intersected the contours. The spatial discretization was picked to be 5 points per mm for each of the contours. This discretization value corresponds to approximately 6-7 points per wavelength for the 1.2 MHz sonications and 5-6 points per wavelength for the 1.44 MHz sonications. These more finely discretized contours were obtained by interpolating and then smoothing the contours manually segmented from the MR images. The skin-gelpad and gelpad-oil contours were derived from the smoothed skin-fat interface as described in the section titled 'Simulation Geometry, Patient Anatomy, and Coregistration'. In the phantom simulations, the phantom-membrane contour separating the phantom from the oil that the transducer is immersed in was manually defined/determined, followed by coregistration, interpolation and smoothing as was described for the manually segmented anatomical contours for the patient simulations.

The pressure simulation calculation volume was chosen to have dimension of approximately 7 by 7 by 9 cm and was centered about the treatment cell position with the misregistration correction value added to it. To save computational time, the pressure field was calculated using a multi discretization grid technique (Ellens and Hynynen 2014), beginning with a spatial discretization length of 0.8 mm, which was narrowed to 0.4 mm, and finally to 0.2 mm by interpolating and recalculating interpolated values in regions having a high Laplacian value.

For each trajectory, the absorbed power density field of a target point is first calculated using the targeting parameters of that point on the trajectory. The absorbed power density fields of the remaining points on the trajectory were obtained by rotation. The point of rotation was taken to be the misregistration corrected cell position with an additional lateral shift equal to the amount of lateral displacement of the calculated absorbed power density maximum from its corresponding target point. The rotation of a field calculated within a rectangular volume geometrically introduces points which are not well defined around the boundaries within the periphery of that volume, which were set to zero in all of the absorbed power density fields for the trajectory, resulting in fields trimmed to a cylinder like shape.

Thermal simulations

The temporal discretization dt was set to 12.5 ms and the spatial discretization used was 0.2 mm. The temperature simulation volume was chosen to be coterminous with the ultrasound volume, and was padded with additional voxels that were modeled to have constant temperature. The padding was removed after the simulation. An initial temperature condition of 37 degrees Celsius was assumed for the entire volume. A constant temperature boundary condition was implemented by holding the outermost two voxels on each face of the padded volume at fixed temperature. On each timestep, for each voxel that was not held at constant temperature, the finite difference calculation used two spatial neighbors on both sides of the voxel in each of the three grid directions.

Whichever media from the layered model were within the thermal simulation volume, were taken into account using tissue parameters of table 1. The thermal dose was calculated at each time step and perfusion was set to zero once the dose reached 240 equivalent minutes at 43 °C. Temperature rise was not simulated in oil meaning that any voxels consisting of oil were kept at constant temperature.

Trajectory point switching time of 50 ms, and the order of sonication of points on the trajectory used were derived from the treatment planning system configuration files. The durations of sonication trajectories were approximated by parameters in the treatment planning system log files and clinical treatment reports. A cooling simulation was performed having duration approximately from the end of sonication to the end of scanning that was derived from timestamps within the log files.

MR-thermometry simulation

The MR-thermometry simulation was done by first converting the simulated temperature values in the simulation grid having 0.2 mm discretization to phases via $\Delta\varphi = \alpha_{PRF}\gamma B_0 TE \Delta T$ where α_{PRF} is the temperature sensitivity coefficient, γ is the gyromagnetic ratio, B_0 is the magnetic field strength and **TE** is the echo time. Next, a temperature value for each MR-thermometry voxel was obtained by calculating the temperature using the same equation, but from the phase value of the average of the complex proton signal within that voxel. The numerical values of $\alpha_{PRF} = -0.0094$ ppm °C $^{-1}$, $\frac{\gamma}{2\pi} = 42.58$ MHz Tesla^{-1}, $B_0 = 3$ Tesla, and $TE = 16$ ms were used. For each sonication, a coronal stack and sagittal slice were placed at the treatment cell position. Another analysis was also carried out in which a coronal stack and sagittal slice were placed at the treatment cell position with the misregistration corrected value added to it. Unless otherwise stated, results shown are performed with the coronal stack and sagittal slice placed at the treatment cell position. The word ‘placed’ in this context means the following: In the case of the coronal stack ‘placed at the treatment cell position’ means that the center of the central voxel of the second coronal slice is placed at the treatment cell position. In the case of the sagittal slice ‘placed at the treatment cell position’ means that the voxel grid of the sagittal slice is defined in such a way that a voxel of the slice has its center at the treatment cell position (this voxel might not be the central voxel of the slice in the sense that the spatial extent of the slice in the anterior posterior direction with respect to the treatment cell position may be asymmetric due to the size of the simulation volume). The sampling interval of the simulated MR-slices was set to 2.57 s to coincide with the sampling interval of slices acquired during treatments. The voxel dimensions of the slices were set to 2.083 by 2.083 by 7 mm to match the ones used in the clinical slices.

Comparison of Clinical Data to Simulation Results:

To assess the accuracy of the simulations pertaining to magnitude of temperature rise, two metrics were used: peak temperature curves as a function of time evaluated using the sagittal slice of simulation

and of treatment as well as thermal dose threshold volume sizes. The $240EM_{43}$ focal volume thermal dose distribution sizes were compared by comparing the $240EM_{43}$ thermal dose focal volumes obtained via voxel summation of clinical and simulated coronal slices. The overlapping information in the sagittal slice is not used in the evaluation of thermal dose: There is only one sagittal slice (7mm thick) which is not thick enough to cover a treatment cell having a 12mm diameter, furthermore the voxels (having already undergone spatial averaging) of the sagittal slice are oriented perpendicular (7mm in LR direction) to those of the coronal slices (7mm in AP direction). When presenting data involving averages of many sonications, the data is presented in relative quantities for purposes including power normalization as well as ease of presentation. However, several examples involving these metrics are presented using absolute quantities as they have the advantage of providing additional information thus enabling presentation of more informative concrete examples of clinical relevance (e.g. peak temperature curve).

Temporal registration of clinical and simulated MR-thermometry slices

Peak temperature time curves calculated from the clinical and simulated sagittal MR-thermometry slices were compared. The first simulated MR-thermometry slice was simulated to occur at approximately 2.57 seconds after the beginning of sonication, whereas the first sagittal acquisition of a sonication of a patient treatment occurred at some time before 2.57 seconds after sonication. The sampling interval between slices was approximately 2.57 seconds for both simulated and clinical slices. However, as a practical matter, on the figures where temperature is represented as a function of time, the zero value of the time axis is set to the beginning of the simulated sonication and the treatment sagittal dynamics are temporally registered (shifted in time) in such a way that the first temperature acquisition is set to occur at t=0, i.e. The first treatment temperature acquisition dynamic is set to have a time value of 0 seconds so that simulated and treatment temperature curves of any given sonication have the same set of time coordinates.

Verification of Transducer Element Phase Calculation

To verify that the transducer element initial velocity phases were appropriately calculated via backpropagation using the targeting parameters, pressure field simulations were ran in homogenous media with initial velocities of transducer elements having the negatives of the transducer element velocity phases in generator reports printed using the Sonalleve system software during the phantom experiment and with initial velocities calculated using the targeting parameters for three sonications. Sonications with three different values of electronic steering were simulated. These sonications were performed at three different depths. The treatment cell positions were selected to be approximately 1 cm apart from one another in depth. For these sonications, the transducer position was approximately the same (varied by less than 1 mm in each of three directions), and the electronic steering value in the depth direction used in the targeting parameters ranged from 4 mm in the anterior direction to 20.9 mm in the posterior direction. When the results of each simulated sonication using the two aforementioned initial velocities were compared, in each case the locations of the absorbed power density maxima coincided, the magnitudes of the absorbed power density maxima differed by less than 1 % and the linear absorbed power density profiles through the absorbed power density maximum along the three axes were in good agreement (see supplementary figure S1) suggesting that the backpropagation calculation using the targeting parameters generated transducer element phases that yield practically the same acoustic fields as the phases used to drive the transducer during the experiment.

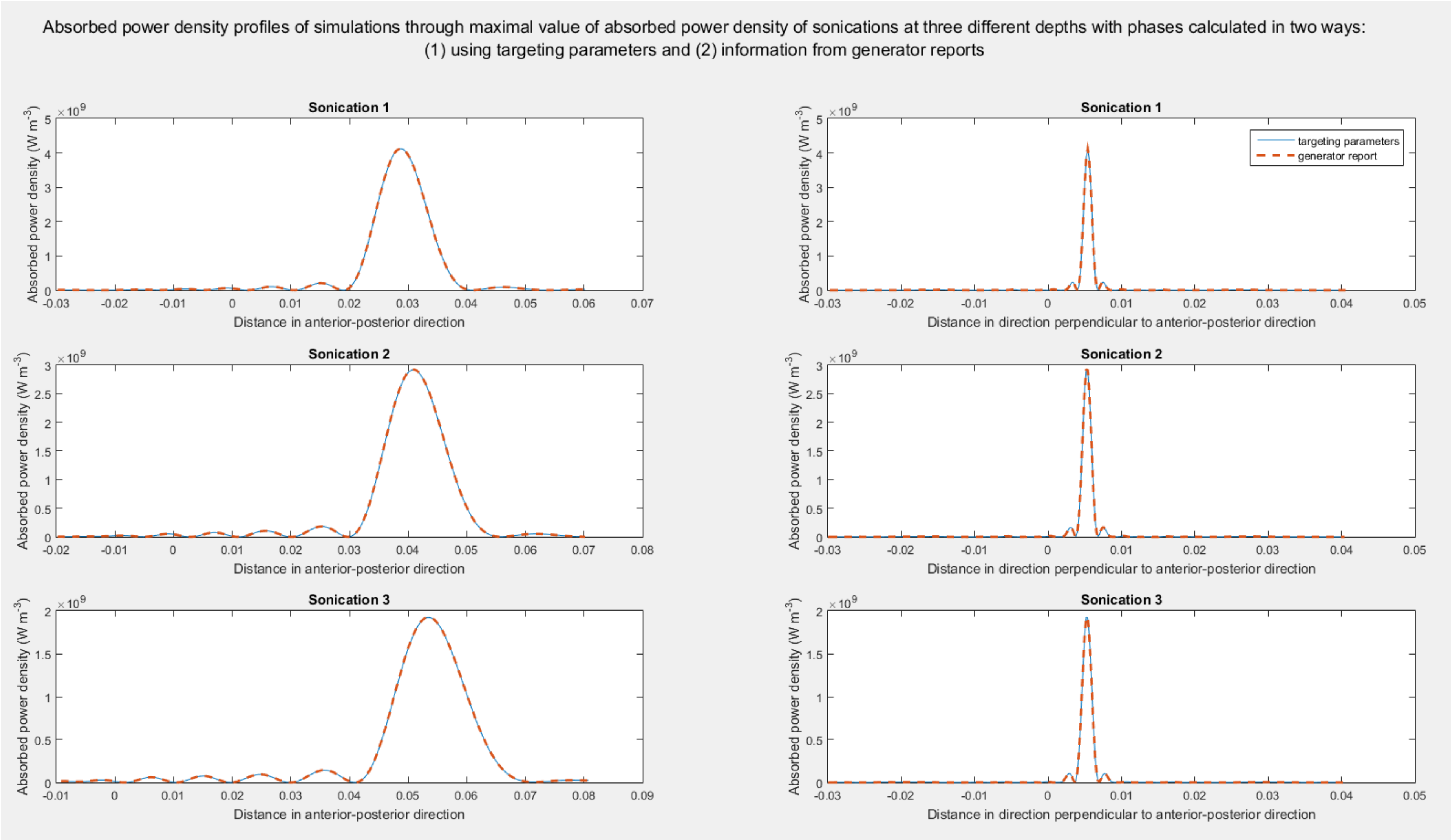

Figure S 1 The linear absorbed power density profiles through the absorbed power density maximum along the anterior-posterior axes and an axis perpendicular to the anterior posterior axis. The units of all horizontal axes are in meters.

Effect of variation of perfusion on center of mass of thermal dose distribution

When comparing the anterior-posterior location of the center of mass of a thermal dose distribution of the sagittal slice of a sonication simulated using a value of perfusion of 1.89 kg($m^{-3}s^{-1}$) to that of a higher value the results are the following: regarding the N = 20 sonications simulated using a fibroid absorption value of 8.6 Np($m^{-1}MHz^{-1}$) in which perfusion was varied to take on the values of 1.89, 10 and 100 kg($m^{-3}s^{-1}$), changing omega from 1.89 to 10 kg($m^{-3}s^{-1}$) yielded displacements ranging from 0 to 0.3 mm towards the posterior direction, and changing omega from 1.89 to 100 kg($m^{-3}s^{-1}$) yielded displacements ranging from 0.7 mm towards the anterior direction to 1.4 mm towards the posterior direction; regarding the N=2 sonications simulated using a fibroid absorption value of 1.2 Np($m^{-1}MHz^{-1}$) in which perfusion was varied to take on the values of 1.89 and 10 kg($m^{-3}s^{-1}$), changing omega from 1.89 to 10 kg($m^{-3}s^{-1}$) yielded displacements of less than 0.1 mm; regarding the N=5 sonications simulated using a fibroid absorption value of 1.2 Np($m^{-1}MHz^{-1}$) in which perfusion was varied to take on the values of 1.89, 10 and 100 kg($m^{-3}s^{-1}$), changing omega from 1.89 to 100 kg($m^{-3}s^{-1}$) yielded displacements ranging from 8.3 mm to 44.8 mm towards the anterior direction and changing omega from 1.89 to 10 kg($m^{-3}s^{-1}$) yielded displacements ranging from 0.2 mm to 6.1 mm towards the anterior direction.

In some sonications, including some of the aforementioned sonications for which perfusion was varied, simulations utilizing the fibroid absorption value of 1.2 Np($m^{-1}MHz^{-1}$) had a thermal dose center of mass on the sagittal slice that based on numerical value appears to be displaced further anteriorly from the non-simulated thermal dose center of mass than those of the simulations utilising larger values of absorption. This apparent displacement at least in the case of some of the aforementioned sonications for which perfusion was varied, is likely not due only to properties of the thermal dose distribution of the focal volume, but likely instead is caused by a lack of heating to such an extent in the focal volume that the thermal dose distribution of the nearfield of the simulated sagittal slice largely contributes to the apparent displacement. The same phenomenon due to lack of focal heating may apply for simulations utilizing a fibroid absorption value of 18 Np($m^{-1}MHz^{-1}$) as well. Visual inspection of the last simulated sagittal thermal dose distribution slice of the five sonications simulated using a fibroid absorption value of 1.2 Np($m^{-1}MHz^{-1}$) in which perfusion was varied to take on the values of 1.89 , 10 and 100 kg($m^{-3}s^{-1}$) revealed that the center of mass of the thermal dose due to focal volume heating of each simulated sonication appeared to be in approximately the same location regardless of the value of perfusion, while the values of the focal volume thermal dose distribution became closer in magnitude to that of the nearfield thermal dose distribution as perfusion was increased (Supplementary Figure S2). Regarding these five sonications, when comparing the location of the center of mass of a thermal dose distribution of the sagittal slice of a sonication simulated using a value of perfusion of 1.89 kg($m^{-3}s^{-1}$) to that of a higher value the resulting displacement values were large at least for the highest value of omega (see previous paragraph). These observations support the aforementioned hypothesis that for a sonication, a case where simulated focal volume heating is low enough to yield a focal volume thermal dose distribution comparable in magnitude to that of the nearfield, can yield a sagittal slice thermal dose distribution center of mass showing a large difference when compared to the center of mass of a simulated sagittal slice thermal dose distribution showing high focal volume heating relative to the nearfield while the locations of the region containing locally high values of thermal dose due to focal heating are approximately the same in both simulated sagittal slice distributions. This effect could be further analysed by recalculating the center of mass in a cropped section of the sagittal simulated MR-thermometry slice instead of the full simulated slice, in which case the aforementioned nearfield heating contribution to the shift would likely be greatly reduced.

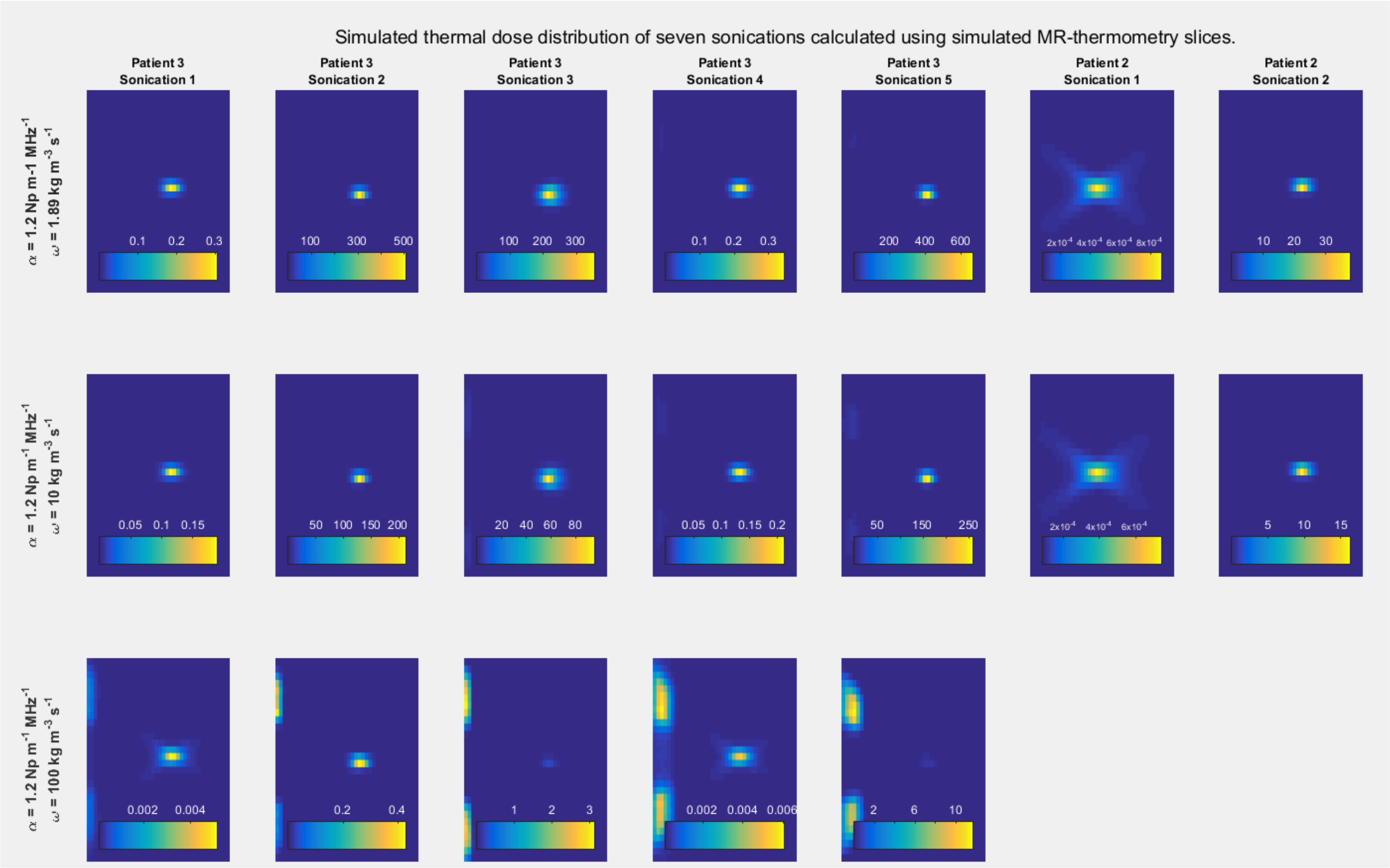

Figure S 2 The last simulated sagittal thermal dose distribution slice is shown for seven sonications.The units of magnitude are equivalent minutes at 43 degrees Celsius. This figure illustrates that the center of mass of the locally high region of thermal dose due to focal volume heating of each simulated sonication appears to be in approximately the same location regardless of the value of perfusion, while the values of the focal volume thermal dose distribution became closer in magnitude to that of the nearfield thermal dose distribution as perfusion was increased.

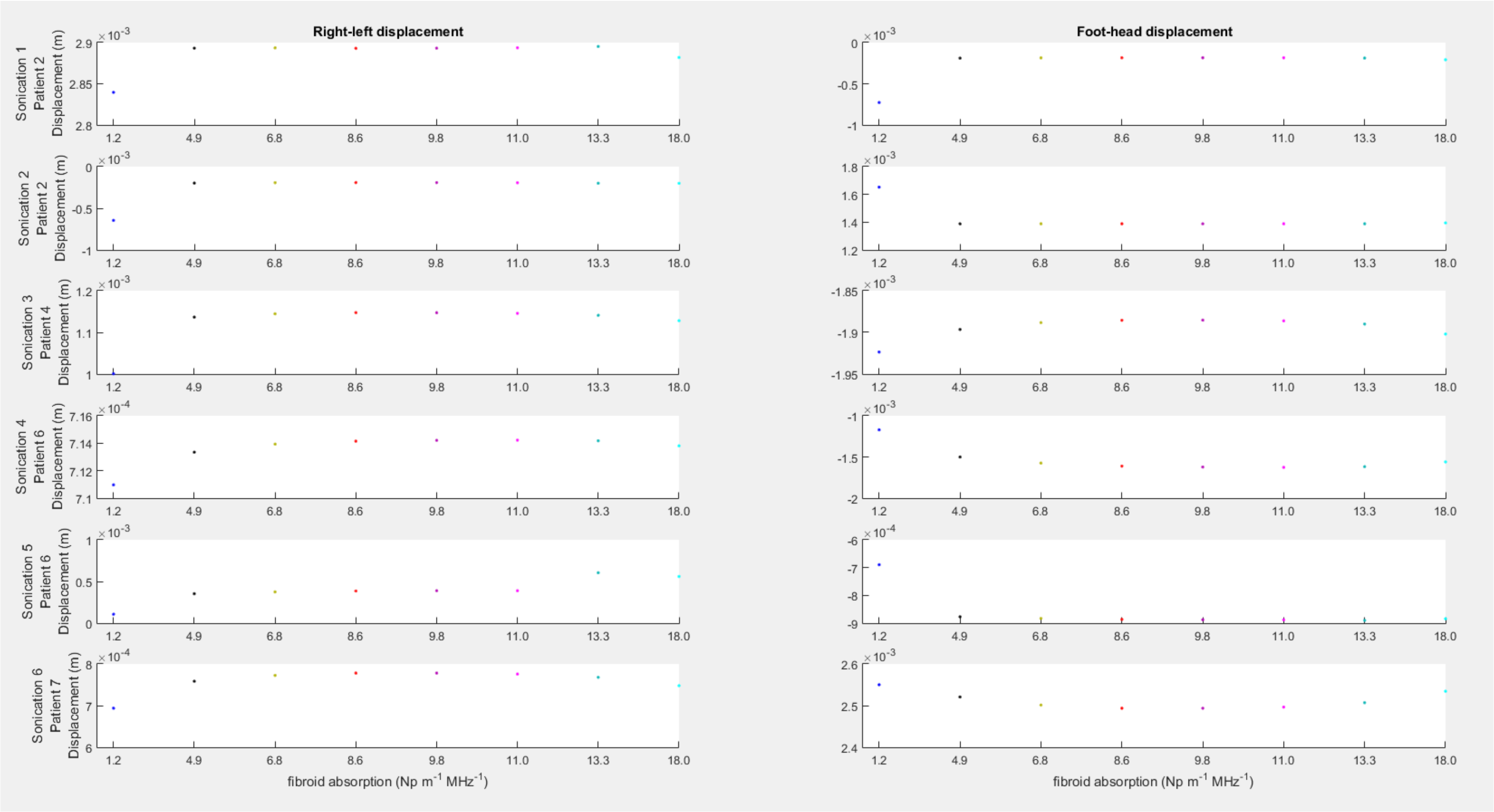

Figure S 3 Displacements between centers of mass of thermal dose distribution of simulation and treatment in the right-left and foot-head directions corresponding to the six sonications for which temperature curves, 240EM$_{43}$ volumes, and anterior-posterior displacements were presented in the manuscript for the absorption values ranging from 1.2 to 18.0 Np(m^{-1}MHz^{-1}).

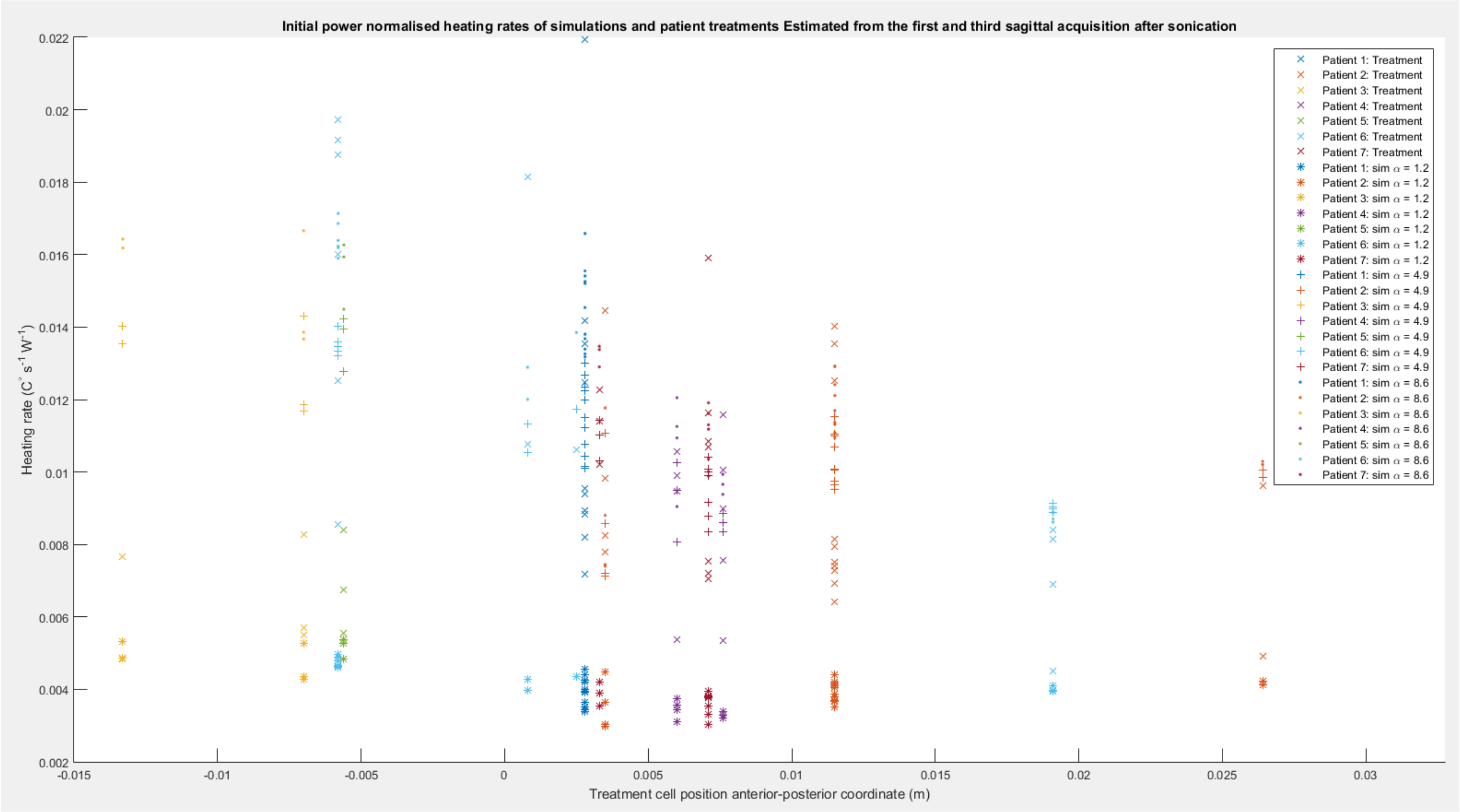

Figure S 4 The idea that intra-patient variation of tissue parameters may be a practically significant contribution to the ranges of discrepancies between peak temperature curves of simulations and treatment is further supported by performing a comparison of the power normalised initial peak temperature heating rates of simulations and treatment. For example, within some patients there are sonications having treatment cell positions with the same depth coordinate that exhibit a range of patient treatment peak initial heating rate results that is roughly equal to the range of analogous results of simulations when varying alpha between 1.2 and 8.6 Np($m^{-1}MHz^{-1}$). The simulated heating rates in this figure were estimated using the difference of the peak temperature of the second simulated sagittal MR-thermometry acquisition and the baseline temperature of 37 degrees Celsius. This figure also illustrates the low degree of clinical heating exhibited by patient 3. In addition, of the 67 sonications considered in this simulation study, the power normalised initial heating rate estimated using the peak temperature in the focal volume of the first and third sagittal MR-thermometry slice acquisitions averaged over sonications was on average lower for patient 3 compared to the other patients, despite the sonications of patient 3 having treatment cell position depth coordinates that were similar to as well as anterior of those of sonications exhibiting a higher degree of heating based on the aforementioned metric that were performed in the treatment of another patient.

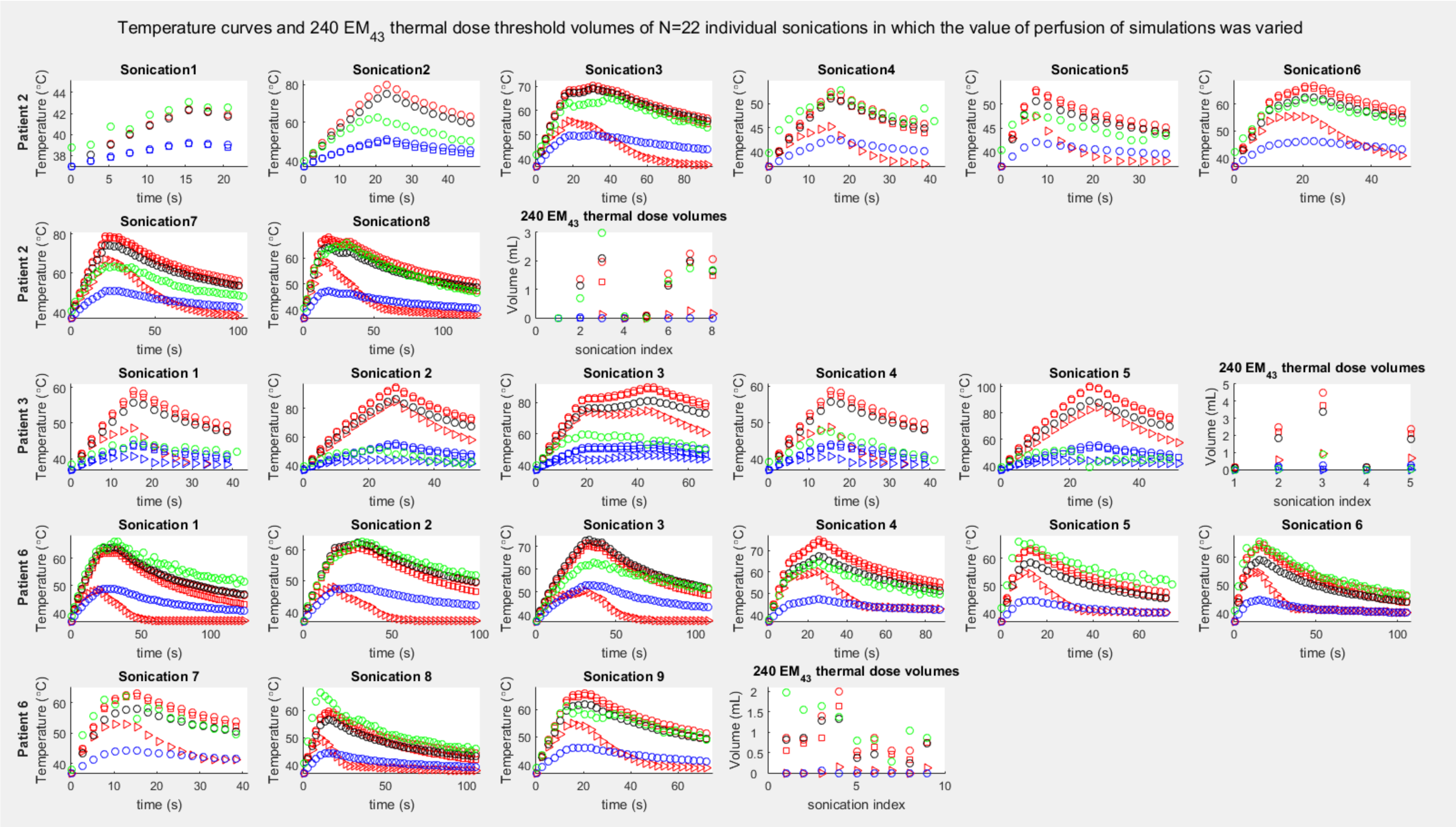

Figure S 5 Peak temperature of the sagittal MR-thermometry slice as a function of time as well as 240EM$_{43}$ thermal dose threshold volumes for simulation and treatment are shown for N=22 sonicaitons in which the perfusion parameter was varied to take on more than one value. The green circle denotes patient treatment. The blue, black, and red colors each represent simulations utilizing an absorption coefficient of 1.2, 4.9, and 8.6 Np(m^{-1}MHz^{-1}) respectively where the circle, square, and triangle denote perfusion values of 1.89, 10, and 100 kg(m^{-3}s^{-1}) respectively. The indexing of sonications in this figure is independent of the indexing elsewhere in the manuscript and supplementary section. The perfusion parameter value of 100 kg(m^{-3}s^{-1}) might be too high to be representative of the patient perfusion in many of the simulated sonications in which it was utilised since the peak temperature curves of the simulations utilizing a fibroid absorption value of 8.6 Np(m^{-1}MHz^{-1}) and this value as a perfusion parameter typically exhibited a high degree of cooling larger than that of the peak temperature curves of patient treatment. Sonication 8 of patient 2 demonstrates good agreement with a perfusion parameter of 10 kg(m^{-3}s^{-1}) where varying the perfusion parameter in simulations from 1.89 to 10 kg(m^{-3}s^{-1}) did not have a strong effect on peak temperature curves, but the effects on the 240EM$_{43}$ thermal dose threshold volumes are substantial.

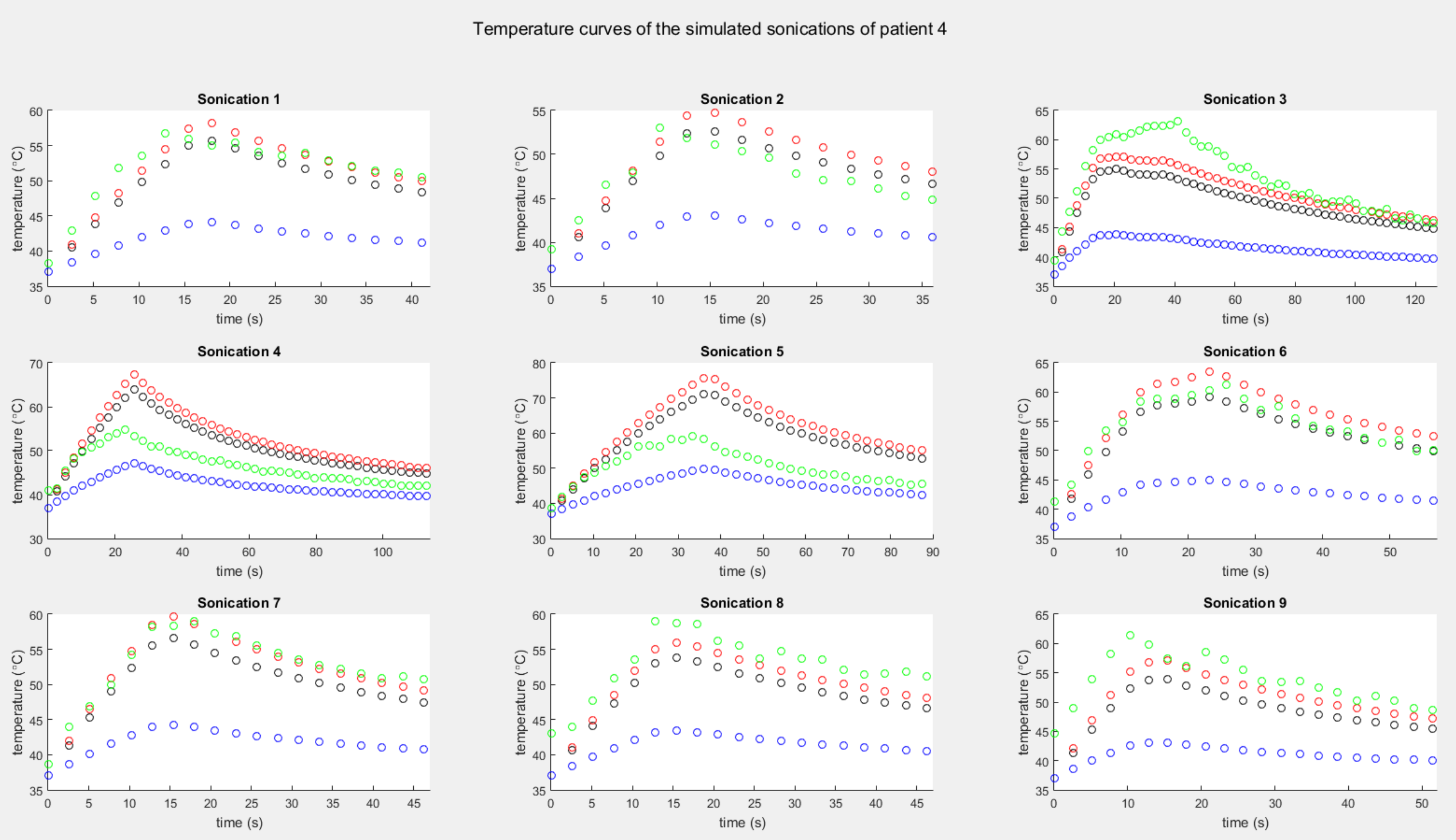

Figure S 6 Peak temperature-time curves of the sagittal MR-thermometry slice of simulations as well as those of the patient treatment are shown for the nine sonications included in the simulation dataset from the sonications performed in the treatment of patient 4. The green curves denote patient treatment. The blue, black, and red colors each represent simulations utlizing a fibroid absorption coefficient of 1.2, 4.9, and 8.6 Np($m^{-1}MHz^{-1}$) respectively. Note that the results in this figure involving simulations utilising values of fibroid absorption of 4.9 and 8.6 Np($m^{-1}MHz^{-1}$) show substantially more heating than those of treatment for sonications 4 and 5 while the other sonications do not exhibit a comparable extent of high simulated heating relative to treatment for the aforementioned values of absorption. These two sonications were performed on the same treatment cell position located on or close to the border/septum of the fibroid.

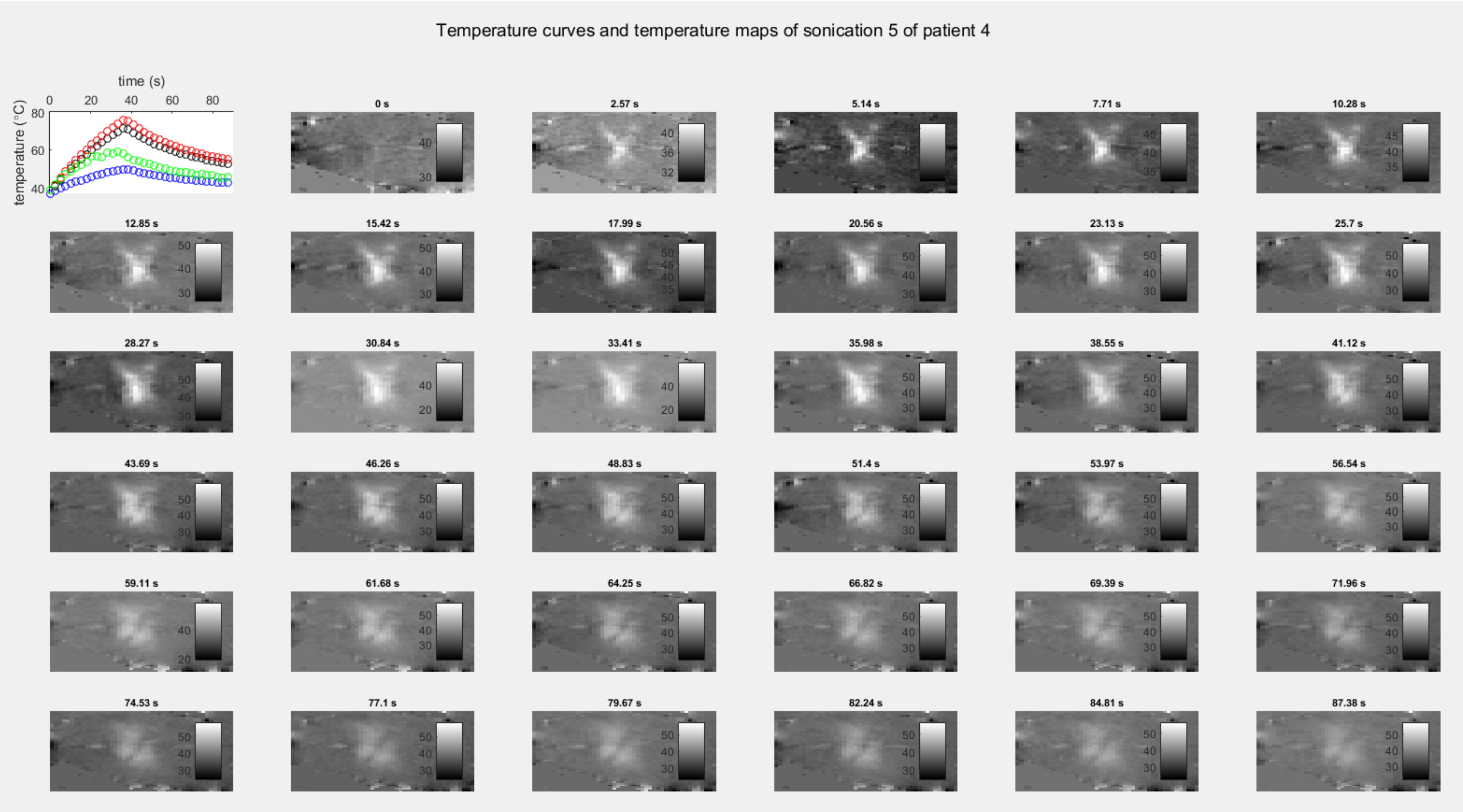

Figure S 7 Peak temperature-time curves of the sagittal MR-thermometry slice of simulations as well as those of the patient treatment are shown in the top-left corner for one of two sonications showing substantially more heating in simulations utlizing fibroid absorption values of 4.9 and 8.6 Np(m^{-1}MHz^{-1}) relative to treatment than the remainder of the sonications included in the simulation dataset from the sonications performed in the treatment of patient 4. The temperature maps of a cropped section of the MR-thermometry slice of patient treatment are shown for timepoints denoting time after begining of simulated sonication which correspond to the time values of the temperature curves. The cropped region shown in these images, while larger than the manually cropped region used to evaluate the peak temperature curve shown for the patient treatment, illustrates a region of cooling shaped like a vessel within the region of the focal volume heating.

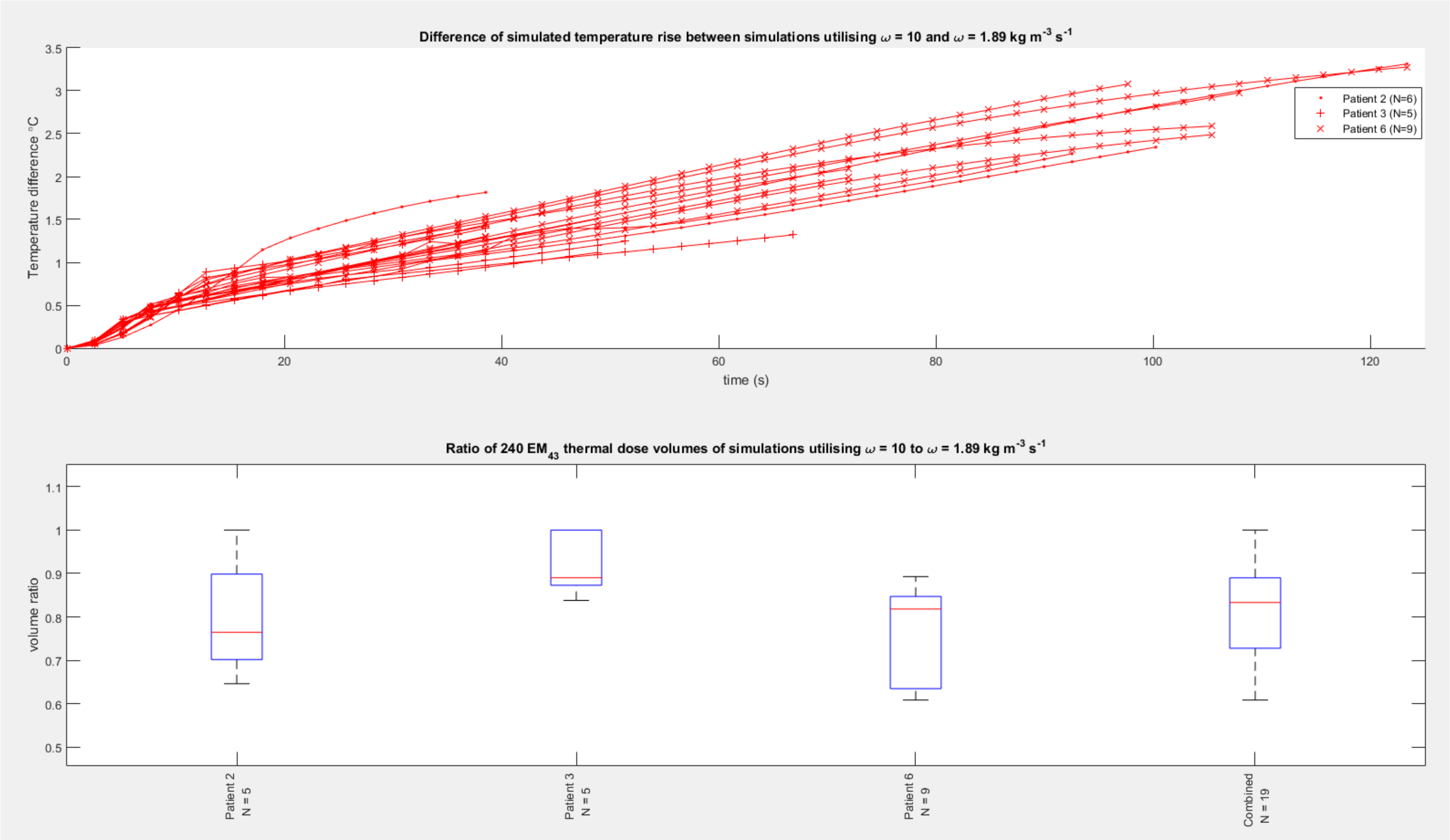

Figure S 8 Effects of varying perfusion on temperature rise and 240EM$_{43}$ thermal dose volumes are shown for N=20 sonications simulated using a fibroid absorption value of 8.6 Np(m^{-1}MHz^{-1}): the top panel shows for each sonication a curve representing the difference obtained by subtracting the peak temperature rise of the sagittal MR-thermometry slice of a simulation utilising a perfusion parameter of 10 kg(m^{-3}s^{-1}) from that of a simulation utilising a perfusion parameter of 1.89 kg(m^{-3}s^{-1}); and the bottom panel shows boxplots of the ratio of the 240EM$_{43}$ thermal dose volume of a sonication obtained via simulation by utilising a perfusion parameter of 10 kg(m^{-3}s^{-1}) to that of the same sonication obtained via simulation by utilising a perfusion parameter of 1.89 kg(m^{-3}s^{-1}). There is data for 19 sonications in the bottom panel because one of the six sonications of patient 2 had a simulated 240EM$_{43}$ thermal dose threshold volume of zero for both of the aforementioned values of perfusion, thus yielding an undefined ratio. This figure shows that while varying the perfusion parameter from 1,89 to 10 kg(m^{-3}s^{-1}) does not have a large effect on peak temperature curves, the effect on 240EM$_{43}$ thermal dose threshold volumes can still be substantial.

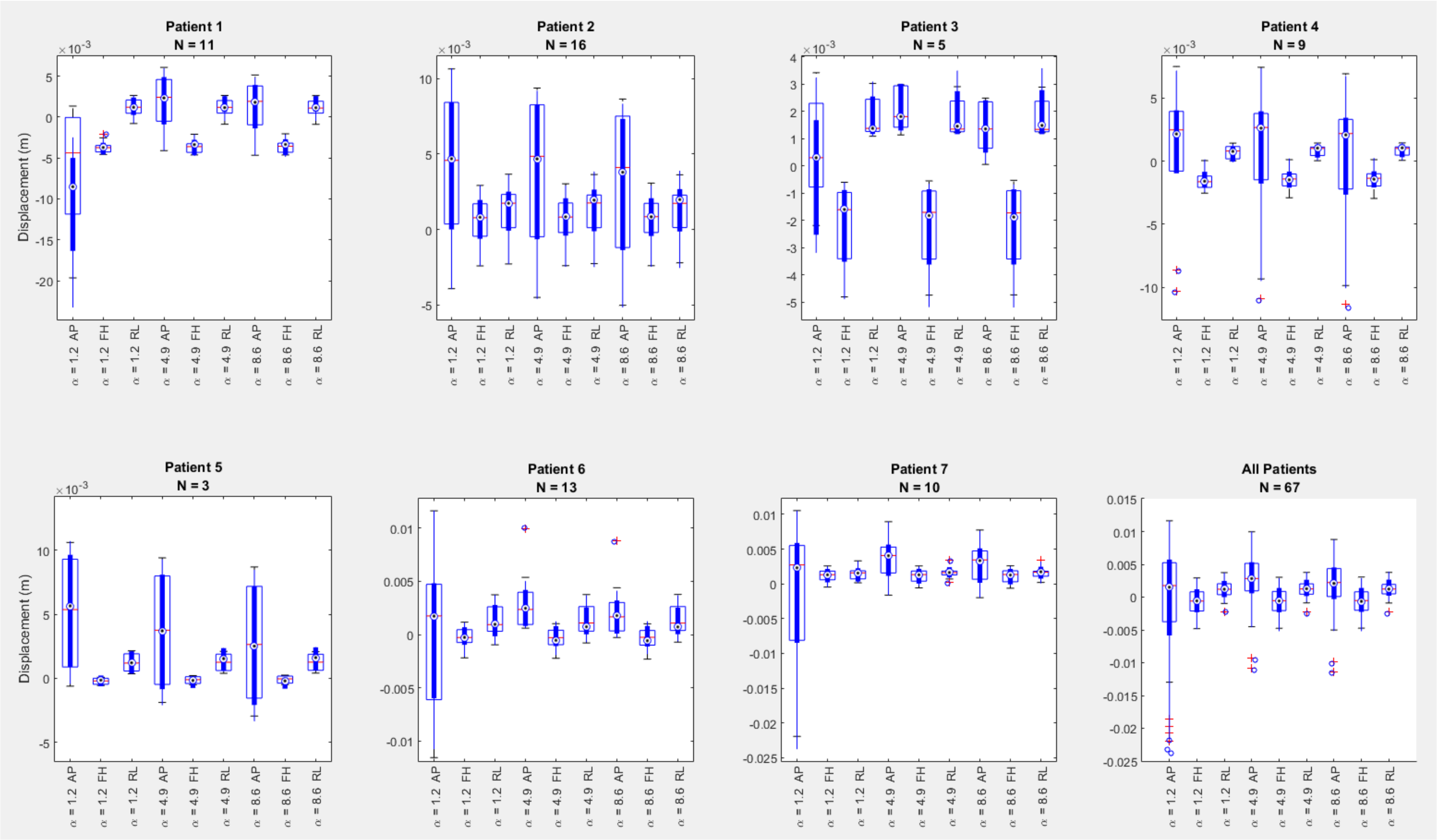

Figure S 9: The coordinate wise differences between the simulated and measured thermal dose center of mass are shown for N=67 sonications using the solid blue narrow boxplots with outliers shown using blue circles. For example, a positive value for an AP displacement means that the simulation of a sonication resulted in a thermal dose center of mass posterior of the treatment. The corresponding results of the analysis in which the simulated sagittal MR-thermometry slice and simulated stack of coronal MR-thermometry slices was placed at the treatment cell position with the misregistration correction added to it are shown using the wide hollow box plots with outliers shown using red plus signs. The value of absorption used is denoted by α in units of Np($m^{-1}MHz^{-1}$) and the value of perfusion used was ω = 1.89 kg($m^{-3}s^{-1}$).

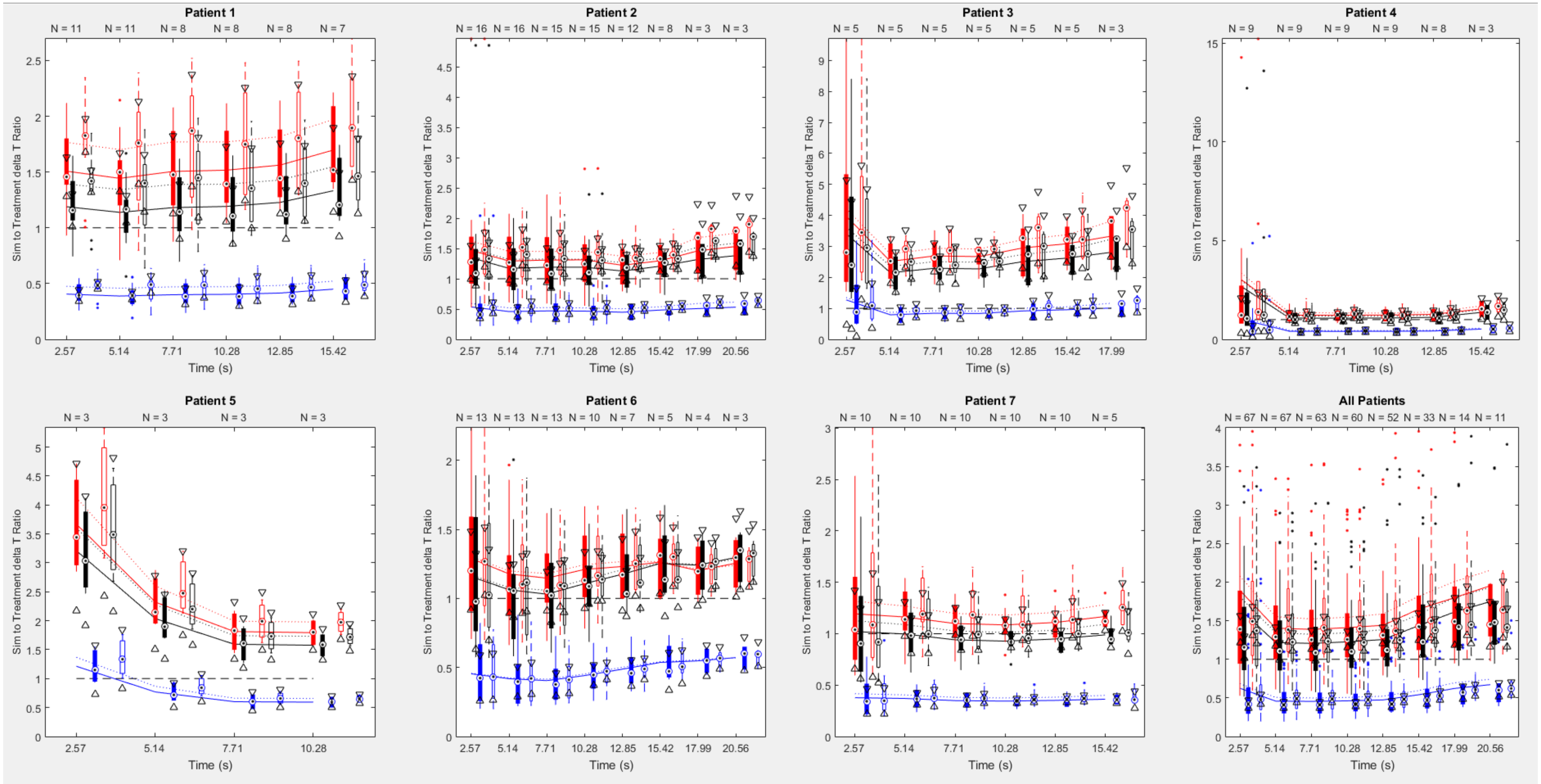

Figure S 10: Ratio of sagittal MR-thermometry peak temperature rise of simulation to patient treatment as a function of time averaged over sonications within individual patient treatments as well as throughout patient treatments is shown using solid lines. The blue, black, and red colors each represent a value of fibroid absorption coefficient of 1.2, 4.9, and 8.6 Np(m^{-1}MHz^{-1}) respectively that was used in simulations. The data is shown only for the durations of the innermost trajectory in order for the averaging to be meaningful (for example averaging a sonication that is cooling while another is heating at a particular timepoint may not be particularly meaningful). The number of sonications is thus variable as a function of time because the sonications do not all have the same innermost trajectory duration. To indicate the degree of discrepancy between simulation and treatment as well as information about the distribution of the data, notched boxplots in solid colors are shown for each timepoint. The triangles represent the notches of the boxplot calculated using the formula $q_2 \pm 1.57\,(q_3-q_1)/(N^{0.5})$ where q_1, q_2, and q_3 are the 25th, 50th, and 75th percentiles respectively and N is the number of observations. The non-overlapping intervals indicated using triangles indicate statistically significant differences of the medians at 5% significance level. The corresponding results of the analysis in which the simulated sagittal MR-thermometry slice was placed at the treatment cell position with the misregistration correction added to it are shown using dotted lines and hollow boxplots. The horizontal constant dashed line having black color denotes unity.

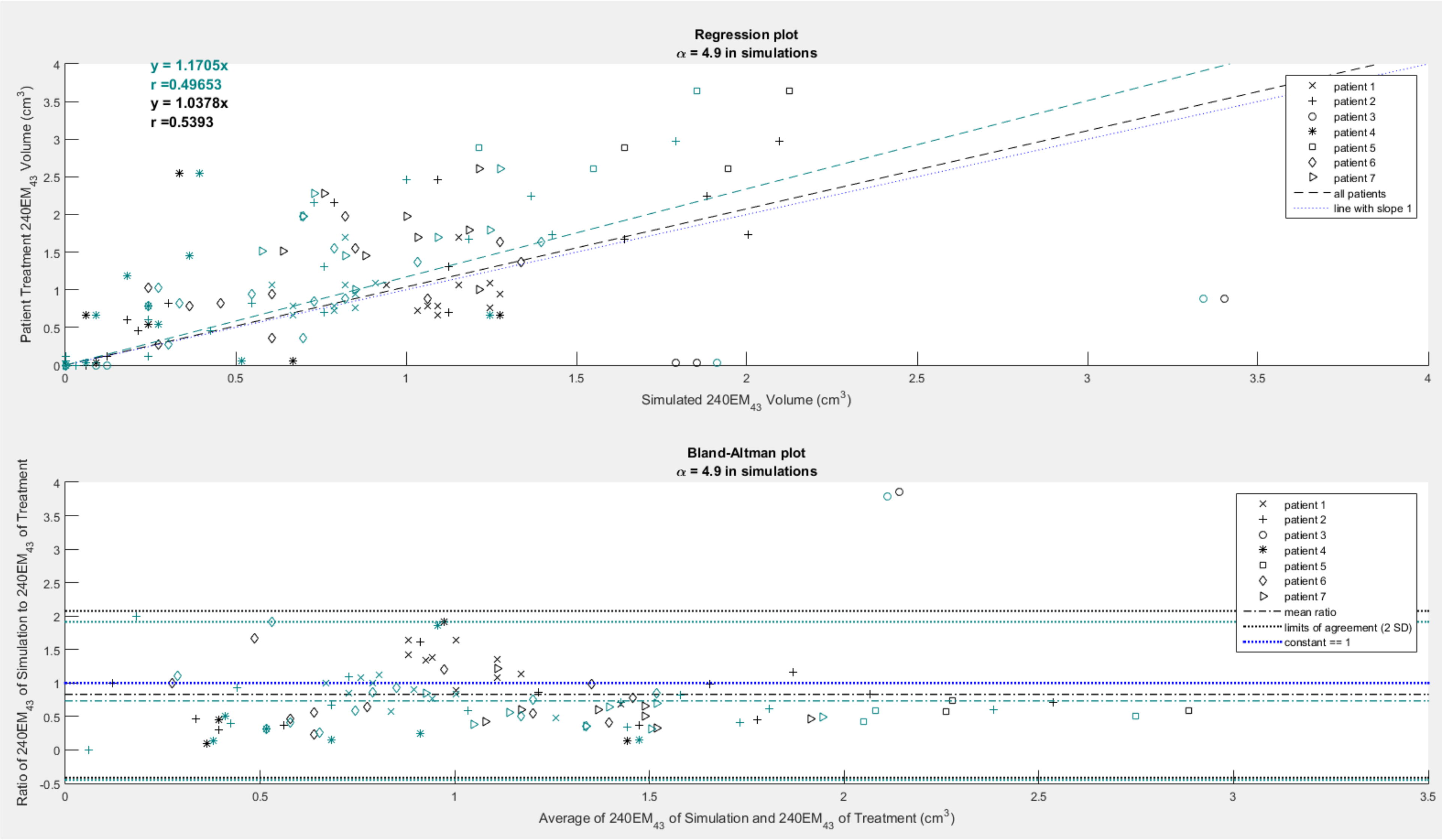

Figure S 11: Thermal dose volume results of the analysis in which the simulated stack of coronal MR-thermometry slices was placed at the treatment cell position are shown in black and the corresponding results of the analysis in which the simulated stack of coronal MR-thermometry slices was placed at the treatment cell position with the misregistration correction added to it are shown in teal color. Top: A linear regression and scatter plot of the $240EM_{43}$ thermal dose volumes of 67 sonications of patient treatments and simulations utilising a fibroid absorption value of 4.9 $Np(m^{-1}MHz^{-1})$. Bottom: A Bland-Altman plot with ratios of $240EM_{43}$ volumes of simulations to treatment as a function of the average of the two quantities of N=54 sonications each having a patient treatment $240EM_{43}$ volume greater than 2 voxels. The limits of agreement are mean ratio ± 2 standard deviations.

Supplementary $240EM_{43}$ thermal dose volume data

The three sonications in which the patient treatment $240EM_{43}$ volume was one voxel had $240EM_{43}$ volume values in voxels of 61, 59, and 3 for simulations utilising a fibroid absorption value of 4.9 Np($m^{-1}MHz^{-1}$), and values in voxels of 82, 78, and 7 for simulations utilising a fibroid absorption value of 8.6 Np($m^{-1}MHz^{-1}$). Of the aforementioned sonications, the two showing the highest thermal dose (and thus overprediction) in simulations correspond to patient 3. The two sonications in which the patient treatment $240EM_{43}$ volume was two voxels had $240EM_{43}$ volume values in voxels of 0 and 22 for simulations utilising a fibroid absorption value of 4.9 Np($m^{-1}MHz^{-1}$), and values in voxels of 0 and 30 for simulations utilising a fibroid absorption value of 8.6 Np($m^{-1}MHz^{-1}$). Of the aforementioned two sonications, the one showing a nonzero value (overprediction) of thermal dose in simulations corresponds to patient 4.

The $240EM_{43}$ volumes of simulations utilising a fibroid absorption value of 1.2 Np($m^{-1}MHz^{-1}$) and a perfusion value of 1.89 kg($m^{-3}s^{-1}$) were zero for 60 of the 67 sonications. The non-zero values of $240EM_{43}$ volumes of simulations utilising a fibroid absorption value of 1.2 Np($m^{-1}MHz^{-1}$) of sonications corresponding to patient 3 are 6, 8, and 8 voxels, yielding simulation to patient treatment ratios of 6.00, 0.28, and 8.00 respectively. The other two sonications of patient 3 included in the simulation dataset had a $240EM_{43}$ volume value of zero voxels for both treatment and simulation utilising a fibroid absorption value of 1.2 Np($m^{-1}MHz^{-1}$). The remaining non-zero values of $240EM_{43}$ of simulations utilising a fibroid absorption value of 1.2 Np($m^{-1}MHz^{-1}$) ranged from 1 to 2 voxels, yielded simulation to treatment ratios remaining under 0.044, and were results of four sonications in total corresponding to patients 1, 2, and 6.

Limitations

The layered model serves only as an approximation of the actual patient anatomy which gives rise to several limitations. For example, manually segmenting the contours involved making subjective decisions regarding which layer heterogeneities are to be included within, which may be influenced by user experience. Also, in order to save time computationally the anatomical velocity contours were chosen to be large enough to accommodate the beam using the criteria that the normal vectors from the transducer elements to the focus intersected with all velocity contours. However, a contour of such size does not necessarily take into account the portion of the energy that escapes from the beam, which although likely small could still contribute to heating within the volume of interest. A third example of a limitation related to the layered model is that due to patient anatomy, the model may be more suitable towards sonications that lie more superficially in the fibroid as well as centrally in terms of the foot-head and right-left directions. This is because in such cases the patient anatomy conforms better to the relatively flat layered model thus making it easier to have contours conforming to patient anatomical layers while having sizes large enough to satisfy the aforementioned normal vector criterion. This may lead to a selection bias of sonications with preference towards ones that are located anteriorly as well as centrally in the foot head and right left directions. Thus, the accuracy of the model regarding sonications performed closest to the periphery of the fibroid in particular in the posterior region of fibroids might require further study.

It is also possible that nearfield focusing effects could also shift a portion of the thermal dose volume outside of the volume corresponding to that of the coronal slices in cases of both simulations and treatment. For example, as an illustration involving simulations, the sonication of patient 7 for which simulations were ran for values of fibroid absorption ranging from 1.2 to 18.0 Np($m^{-1}MHz^{-1}$), exhibits a simulated thermal dose center of mass that is beyond 7 mm posterior of that of the patient treatment for most of the values of alpha that were simulated (sonication 6 of figure 7). For this sonication, the simulated $240EM_{43}$ thermal dose volume was recalculated by using a set of simulated coronal slices placed 7 mm posterior of the location of the set of simulated coronal slices indicated in the methods and materials section, which yielded a result larger by a factor of about 1.4 for a fibroid absorption value of 4.9 Np($m^{-1}MHz^{-1}$). To partially overcome this effect, one could instead of only volume, also consider the mean diameter of the coronal slice showing the largest $240EM_{43}$ thermal dose threshold volume as well as the length of the $240EM_{43}$ thermal dose volume on the sagittal slice as in the approach of Kim et al. 2012. Another potential benefit of this latter approach is that volumes are inherently more sensitive to error than single dimensional quantities, and that the single dimensional quantities could provide a more detailed characterisation of the discrepancies.

Several approximations involving the treatment device were made in the implementation of the simulations. The model assumes that the velocity of the transducer elements are uniform, which is likely not the case in reality. This limitation could be overcome by vibrometer measurements and/or back projection techniques (Clement and Hynynen 2000), (Kreider et al 2013). It was also assumed that the transducer was immersed in oil during the power calibration performed by the manufacturer of the Sonalleve which is used to convert electrical power to acoustic power, and that the power emitted from the transducer surface was the same as the absorbed power measured by the brush target used in the aforementioned calibration. These assumptions manifested in the simulations as follows: the attenuation of oil was assigned a value of zero (because under the first assumption the attenuation of the oil is already accounted for in the calibration) and the velocity of the transducer elements was scaled to match the nominal power indicated in the log files of the treatment. Furthermore, electronic steering

based power compensations applied by the Sonalleve clinical system were not taken into account in the simulations. This may have contributed to the underprediction of patient treatment $240EM_{43}$ thermal dose threshold volumes exhibited by the simulations. There is also some uncertainty regarding the relative positioning of the transducer, MR-thermometry slices, and treatment cell position relative to each other involving the misregistration correction, which was partially overcome by incorporating two sets of simulated MR-thermometry slice positions into the analysis of simulated MR-thermometry data (see supplementary figures S9, S10, and S11). In addition it was assumed that the elements of the transducer were oriented towards the geometric focus.

References appearing in supplementary section

Clement GT, Hynynen K. Field characterization of therapeutic ultrasound phased arrays through forward and backward planar projection. *The Journal of the Acoustical Society of America*. 2000;108(1):441-446.

Ellens N, Hynynen K. Simulation study of the effects of near- and far-field heating during focused ultrasound uterine fibroid ablation using an electronically focused phased array: A theoretical analysis of patient safety. *Medical Physics*. 2014;41(7):72902-1-15.

Kim Y, Trillaud H, Rhim H, et al. MR Thermometry Analysis of Sonication Accuracy and Safety Margin of Volumetric MR Imaging–guided High-Intensity Focused Ultrasound Ablation of Symptomatic Uterine Fibroids. *Radiology*. 2012;256(2):627-637.

Kreider W, Yuldashev P, Sapozhnikov O, et al. Characterization of a multi-element clinical HIFU system using acoustic holography and nonlinear modeling. *IEEE Transactions on Ultrasonics, Ferroelectrics, and Frequency Control*. 2013;60(8):1683-1698
